# CoronaVR: A Computational Resource and Analysis of Epitopes and Therapeutics for Severe Acute Respiratory Syndrome Coronavirus-2

**DOI:** 10.3389/fmicb.2020.01858

**Published:** 2020-07-31

**Authors:** Amit Kumar Gupta, Md. Shoaib Khan, Shubham Choudhury, Adhip Mukhopadhyay, Sakshi, Amber Rastogi, Anamika Thakur, Pallawi Kumari, Manmeet Kaur, Shalu, Chanchal Saini, Vandna Sapehia, Barkha, Pradeep Kumar Patel, Kailash T. Bhamare, Manoj Kumar

**Affiliations:** ^1^Virology Unit and Bioinformatics Centre, Institute of Microbial Technology, Council of Scientific and Industrial Research (CSIR), Sector 39-A, Chandigarh, India; ^2^Academy of Scientific and Innovative Research (AcSIR), Ghaziabad, India

**Keywords:** SARS-CoV-2, 2019-nCoV, COVID-19, epitopes, therapeutics, primers

## Abstract

In December 2019, the Chinese city of Wuhan was the center of origin of a pneumonia-like disease outbreak with an unknown causative pathogen. The CDC, China, managed to track the source of infection to a novel coronavirus (2019-nCoV; SARS-CoV-2) that shares approximately 79.6% of its genome with SARS-CoV. The World Health Organization (WHO) initially declared COVID-19 as a Public Health Emergency of International Concern (PHEIC) and later characterized it as a global pandemic on March 11, 2020. Due to the novel nature of this virus, there is an urgent need for vaccines and therapeutics to control the spread of SARS-CoV-2 and its associated disease, COVID-19. Global efforts are underway to circumvent its further spread and treat COVID-19 patients through experimental vaccine formulations and therapeutic interventions, respectively. In the absence of any effective therapeutics, we have devised h bioinformatics-based approaches to accelerate global efforts in the fight against SARS-CoV-2 and to assist researchers in the initial phase of vaccine and therapeutics development. In this study, we have performed comprehensive meta-analyses and developed an integrative resource, “CoronaVR” (http://bioinfo.imtech.res.in/manojk/coronavr/). Predominantly, we identified potential epitope-based vaccine candidates, siRNA-based therapeutic regimens, and diagnostic primers. The resource is categorized into the main sections “Genomes,” “Epitopes,” “Therapeutics,” and Primers.” The genome section harbors different components, viz, genomes, a genome browser, phylogenetic analysis, codon usage, glycosylation sites, and structural analysis. Under the umbrella of epitopes, sub-divisions, namely cross-protective epitopes, B-cell (linear/discontinuous), T-cell (CD4^+^/CD8^+^), CTL, and MHC binders, are presented. The therapeutics section has different sub-sections like siRNA, miRNAs, and sgRNAs. Further, experimentally confirmed and designed diagnostic primers are earmarked in the primers section. Our study provided a set of shortlisted B-cell and T-cell (CD4^+^ and CD8^+^) epitopes that can be experimentally tested for their incorporation in vaccine formulations. The list of selected primers can be used in testing kits to identify SARS-CoV-2, while the recommended siRNAs, sgRNAs, and miRNAs can be used in therapeutic regimens. We foresee that this resource will help in advancing the research against coronaviruses.

## Introduction

The world is currently undergoing and living with the great threat of pathogenic severe acute respiratory syndrome coronavirus-2 (SARS-CoV-2), which has newly emerged from Wuhan, Hubei province, China (Du Toit, 2020; Hui et al., 2020; Wang C. et al., 2020). Apart from this, in recent years, we have also witnessed sporadic outbreaks and epidemics of various lethal viruses, i.e., Ebola, Zika, Nipah, etc (Gupta et al., 2016, 2020). The current pandemic of SARS-CoV-2 (also named as 2019-nCoV) is now reported to spread over 199 countries and to be responsible for excessive economic loss worldwide (Zhang and Liu, 2020). The World Health Organization (WHO) declared it a public health emergency with a global alert (Ryu and Chun, 2020; Wang C. et al., 2020). Overall, more than 10 million cases and over 0.5 million deaths had been reported worldwide by the end of June 2020^1^. Earlier in different years, CoVs have emerged periodically in various regions worldwide with different death rates (Ksiazek et al., 2003; Bogoch et al., 2020; Guarner, 2020). During the epidemic in 2002–2003, severe acute respiratory syndrome coronavirus (SARS-CoV) led to reported deaths and infected cases of 916 and 8422, respectively. Likewise, another outbreak of Middle East respiratory syndrome coronavirus (MERS-CoV) was reported in 2012, with 543 deaths out of 1401 total cases, giving it a mortality rate of around ∼39% (de Wit et al., 2016).

Coronaviruses (CoVs) are positive-sense single-stranded enveloped RNA viruses belonging to the *Coronaviridae* family (The, 2020). CoVs are the largest known RNA virus genomes, being 27 to 32 kb in length. CoV genomes contains 10–12 open reading frames (ORFs) that encode for the four structural proteins, i.e., surface glycoprotein (or spike) (S), envelope (E), membrane glycoprotein (M), and nucleocapsid (N), 16 non-structural proteins (NSP1–NSP16) (orf1ab polyprotein), other accessory proteins like ORF3a, ORF6, ORF7a, ORF7b, ORF8, and ORF10. These are only RNA viruses, which encode proofreading machinery, i.e., exonuclease and other replicase proteins, for the regulation of fidelity (The, 2020).

Coronaviruses are genotypically divided into four genera, viz., alpha, beta, gamma, and delta coronaviruses. Among these, beta coronaviruses are further classified into four subgroups, i.e., A, B, C, and D (Lu et al., 2020). Previously, six CoVs, two from the alpha group (HCoV-229E and HCoV-NL63) and four belonging to the beta group [HCoV-HKU1 (subgroup-A), HCoV-OC43 (A), SARS-CoV (subgroup-B), MERS-CoV (subgroup-C)], were known to infect humans. SARS-CoV-2 becomes the seventh coronavirus member to infect humans (Cheng and Shan, 2020; Zhu et al., 2020). CoVs are highly pathogenic agents known to cause mainly fatal respiratory ailments (like pneumonia) and to infect various species like humans, bats, pigs, etc (Huang et al., 2020; Lu et al., 2020; Wang C. et al., 2020). Common symptoms are fever, cough, fatigue, breath shortness, muscle ache, headache, diarrhea, etc (Chen et al., 2020; Del Rio and Malani, 2020; Huang et al., 2020; Wu et al., 2020).

Different strategies have been trialed and applied to combat these viruses (Dennis Lo and Chiu, 2020; Maxmen, 2020; Watts et al., 2020; Zhang J. et al., 2020). Primarily, four proteins, which include two proteases, i.e., coronavirus main proteinase (3CLpro) and papain-like protease (PLpro), which are responsible for the proteolysis process, a replicase RNA-dependent RNA polymerase (RdRp) responsible for the replication of RNA genome, and surface glycoprotein (spike), which mediates viral entry and fusion to host cells, are essential for the CoVs, making them preferred targets for therapeutics (Du et al., 2017; Cheng and Shan, 2020; Goo et al., 2020; Morse et al., 2020; Zhang J. et al., 2020). Researchers have mainly explored the ability of existing FDA-approved drugs to control SARS-CoV-2 (Zumla et al., 2016; Lu, 2020). For example, Wang et al., has shown that Remdesivir (GS-5734), a nucleotide prodrug, and Chloroquine effectively inhibit 2019-nCoV *in vitro* (Colson et al., 2020; Wang M. et al., 2020). Remdesivir is known to exhibit broad antiviral activity and has also previously been shown to have effective inhibition efficiency against MERS-CoV, SARS-CoV, Ebola, and Nipah (de Wit et al., 2020; Lu, 2020; Sheahan et al., 2020). Further, various antiviral agents are also in separate clinical trials targeting different SARS-CoV-2 genomic regions/proteins (Maxmen, 2020).

Furthermore, different studies have also reported potential inhibitors to combat CoVs (Momattin et al., 2019; Shen et al., 2019; Totura and Bavari, 2019; Xia et al., 2019). Various studies have also shown the use of different vaccine candidates primarily based on the spike (S), nucleocapsid (N), and envelope (E) proteins (Schoeman and Fielding, 2019; Yong et al., 2019; Zumla et al., 2019; Goo et al., 2020; Tian et al., 2020).

Additionally, various groups have also advocated the use of immune-informatics and computational approaches to target the different proteins of CoVs (SARS as well as MERS). For example, Qamar et al., provide B- and T-cell epitopes against the MERS-CoV spike (S) protein (Tahir Ul Qamar et al., 2019). Srivastava et al., used the *in silico* method to design a multi-epitope vaccine (MEV) against MERS-CoV and SARS-CoV (Srivastava et al., 2018, 2019). Shi et al. (2015) have screened epitope-based vaccine targets against MERS-CoV. Another study provides N protein-based B and CTL epitopes against MERS-CoV (Hori et al., 1989). Recently, a report identified T-cell and B-cell epitopes in the surface glycoprotein of 2019-nCoV (Baruah and Bose, 2020). However, there is no approved drug and licensed vaccine available to combat the virus. Therefore, effective control strategies are urgently required to combat this deadly pathogen (Kickbusch and Leung, 2020; Lu, 2020; Zhang and Liu, 2020). To support the global efforts to fight this virus, we have performed an *in silico* analyses and developed a resource of vaccine candidates and therapeutics to assist the global scientific community.

## Materials and Methods

### Data Collection and Curation

The aim of the current work and analysis is to target all of the Human infecting coronaviruses, with a prime focus on SARS-CoV-2. Complete genome sequences of the CoVs having Humans as hosts were retrieved from the NCBI. An advanced search interface is also deployed on the server to serve the users’ requirements. Along with this, we have also implemented a genome browser for interactive graphical visualization utilizing JBrowse (Buels et al., 2016). Further, as the world is currently suffering from the outbreak of SARS-CoV-2, we have primarily concentrated on the alternative therapeutic options and vaccine candidates. For this, we mainly utilized the protein and gene sequences of the reference SARS-CoV-2 (NC_045512.2). We have also explored the cross targeting and conservancy of different putative regimens against the other six reference CoVs, namely, SARS-CoV (NC_004718.3), MERS-CoV (NC_019843.3), HCoVs NL63 (NC_005831.2), HCoVs 229E (NC_002645.1), HCoVs OC43 (NC_006213.1), and HCoVs HKU1 (NC_006577.2).

### Vaccine Epitopes

**FIGURE 1:**
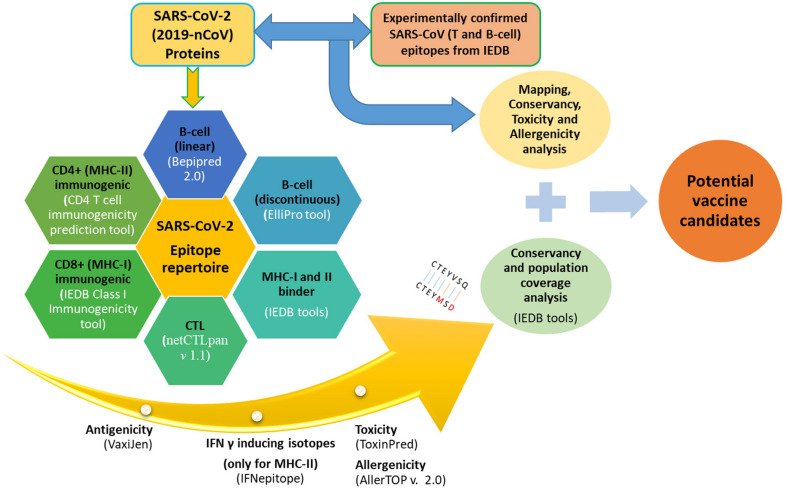
Complete pipeline of epitope prediction and analysis.

The sequences of a large polyprotein (ORF1ab), four structural proteins [Envelope (E), Spike (S), Nucleocapsid (N), and Membrane (M)], and accessory proteins (ORF3a, ORF6, ORF7a, ORF7b, ORF8, and ORF10) of annotated SARS-CoV-2 (NC_045512.2) were retrieved and utilized for the analysis. These sequences were used to predict putative T-cell epitopes (MHC-I and MHC-II binders, Cytotoxic T-lymphocytes (CTL), and Immunogenic CD8^+^ and CD4^+^ epitopes) and B-cell epitopes (linear and conformational) that can be used for designing vaccines against CoVs. An overview of the epitope analysis pipeline is depicted in Figure 1.

### T-Cell Epitope Prediction

We predicted MHC-I and MHC-II binders, CTL epitopes, immunogenic CD8^+^ and CD4^+^ T-cell epitopes, and IFN-γ-inducing peptides (restricted by MHC-II) from all protein sequences of SARS-CoV-2.

For MHC-I and MHC-II binding prediction, we used the corresponding tools available at the Immune Epitope Database (IEDB) Epitope analysis tool page^2^ (Peters and Sette, 2005; Nielsen et al., 2007). For this, the “IEDB recommended” approach was utilized. This approach adopts a consensus method comprising ANN, SMM, and CombLib (if the predictor is available for a particular HLA; otherwise, it uses NetMHCpan EL) for MHC-I and NN-align, SMM-align, CombLib, and Sturniolo for MHC-II (Kim et al., 2012). Shortlisting of predicted binders can be done based on percentile ranks and predicted affinities, where peptides with low percentile rank and low-affinity value (IC50 < 50 nM) are considered good binders (Kim et al., 2012).

The prediction of CD8^+^ (CTL) T-cell epitopes was performed using NetCTLpan *v* 1.1 Server^3^ for 12 HLA supertypes (A1, A2, A3, A24, A26, B7, B8, B27, B39, B44, B58, and B62) (Stranzl et al., 2010). While predicting CTL epitopes, it takes into account various sequence-processing steps such as cleavage by proteasomes, TAP binding, and MHC-I binding (Stranzl et al., 2010). MHC-I-restricted immunogenic peptides were identified using the “IEDB Class I Immunogenicity tool”^4^ with the default settings (Calis et al., 2013). This is based on amino acid properties and their respective positions within the sequence and gives an output in the form of scores, where a higher score indicates a greater probability of eliciting an immune response (Calis et al., 2013).

The immunogenicity of MHC-II restricted peptides was predicted using the “CD4 T cell immunogenicity prediction tool” available at the IEDB^5^. The prediction was performed with the “IEDB recommended” method, which uses a combination of MHC-binding to seven alleles and the immunogenicity method (Dhanda et al., 2018). The output is in the form of a table containing a description of the input sequences along with the combined score, immunogenicity score, 9-mer peptide core, median percentile rank, and score for each of the seven alleles.

Furthermore, Interferon-gamma (IFN-γ) is secreted by T-helper cells and is of central help in clearing the viruses from the host (Chesler and Reiss, 2002). IFN-γ-inducing peptides were predicted among positive MHC-II binders (15-mer) using the IFNepitope web server^6^ (Dhanda et al., 2013). The default settings (“motif and SVM hybrid” and the “IFN-gamma vs. Non-IFN-gamma” model) were used to predict IFN-γ-inducing peptides based on score, where the higher the score, the higher the chance of inducing IFN-γ (Dhanda et al., 2013).

### B-Cell Epitope Prediction

The identification of linear (continuous) B-cell epitopes is an important step in designing a vaccine against a microorganism. Linear B-cell epitope prediction was accomplished using the “BepiPred Linear Epitope Prediction 2.0” method available at the B-cell epitope prediction tool of the IEDB^7^. The tool is based on the random forest algorithm and was trained on amino acids of epitopes and non-epitopes identified from antigen-antibody crystal structures (Jespersen et al., 2017). Amino acid residues with scores greater than the default threshold value of 0.5 are envisaged as being part of an epitope (Jespersen et al., 2017).

The conformational B-cell epitopes are discontinuous or scattered amino acid sequences that make up an antigen and interact with B-cell receptors (BCR) (Sanchez-Trincado et al., 2017). Prediction of these discontinuous B-cell epitopes was performed using the ElliPro tool available at the IEDB^8^ (Ponomarenko et al., 2008). It predicts discontinuous B-cell epitopes based on the 3D structure of protein antigen depending on selected parameters, with the defaults being 0.5 and 6 Angstrom (Å) for minimum score and maximum distance, respectively (Ponomarenko et al., 2008). The output result is in the form of a table displaying “amino acid residues,” “Number of residues,” “Score,” and a link to “3D structure” (Ponomarenko et al., 2008).

### Feature Profiling of Selected B- and T-Cell Epitopes

The shortlisted predicted epitopes (B-cell and T-cell) were analyzed for important features such as antigenicity, toxicity, and allergenicity. The probable peptide-based vaccine epitopes must be antigenic, non-toxic, and non-allergenic.

### Antigenicity Prediction

Antigenicity prediction of the selected epitopes was performed to find the antigenic peptides. To accomplish this, we used the Vaxijen v2.0 server^9^ to predict the antigenicity of these predicted MHC-I and MHC-II binders, CTL epitopes, immunogenic CD8^+^ and CD4^+^ T-cell epitopes, and linear B-cell epitopes. Vaxijen v2.0 was used with a default cut-off of 0.4, indicative of viral antigens, to assess the antigenicity of these peptides (Doytchinova and Flower, 2007).

### Toxicity and Allergenicity Prediction

The toxicity of antigenic B-cell and T-cell epitopes with a Vaxijen score above 0.4 was predicted using the ToxinPred web server^10^ (Gupta et al., 2013). It is based on a quantitative matrix and Support Vector Machine (SVM) utilizing various peptide properties (Gupta et al., 2013). We used the SVM (Swiss-Prot)-based method while keeping all other criteria as default. Epitopes with the prediction result “Non-toxin” were used for further analysis. Likewise, putative vaccine candidates must be checked for allergenicity to prevent allergic responses in the host that may be caused by vaccination (McKeever et al., 2004). We used AllerTOP v. 2.0^11^ to predict the allergenicity of the epitopes being forecasted as “Non-toxic” by ToxinPred. This was developed based on using the k-nearest neighbors (kNN) method to discriminate allergens from non-allergens (Dimitrov et al., 2014).

### Epitope Conservancy Analysis

The conservancy of the predicted epitopes was further analyzed using the epitope conservancy tool available at the IEDB^12^ (Bui et al., 2007). Conservancy is an indication of the percentage identity of the selected epitopes with the proteins of other similar organisms (here, other coronaviruses). We tested the conservancy of predicted epitopes with the other six coronavirus strains that are responsible for causing respiratory illnesses in humans, comprising two alpha coronaviruses (NL63 and 229E) and four beta coronaviruses (SARS, MERS, OC43, and HKU1).

### Population Coverage Analysis

The numerous polymorphic HLAs present in different populations have varied frequencies, and the epitopes restricted by such HLAs would have biased population coverage (Sidney et al., 2010). Hence, during a vaccine design, population coverage must be accounted for to avoid a decrease in the applicability of a vaccine candidate in some populations (Bui et al., 2006). Therefore, it is vital to calculate the frequency of individuals that are anticipated to respond to a given epitope set based on HLA typing (Bui et al., 2006).

We further analyzed the population coverage of the predicted CD8^+^ (MHC-I), CD4^+^ (MHC-II), and CTL epitopes and their respective HLA alleles using the IEDB population coverage tool^13^ (Bui et al., 2006). This reflects the percentage of individuals in a population likely to respond to at least one T-cell epitope from the collection (Bui et al., 2006). The “HLA–epitope pairs” set (epitopes with their restricted HLA alleles) was utilized to compute the projected population coverage (PPC) using query- “area_country_ethnicity” and selecting each of the 16 areas to provide broad global coverage, including China.

### Coronavirus Derived T- and B-Cell Epitopes

The T-cell (MHC class I and class II) and B-cell epitopes of all coronaviruses around the world were searched in the IEDB by querying “Coronavirus” (taxonomy ID: 11118). The search was restricted to “Positive Assays Only” for both “T-cell Assays” and “B-cell Assays” for “Any Host,” “Any MHC restriction,” and “Any Disease.”

### SARS-CoV Derived T- and B-Cell Epitopes

The T-cell (MHC class I and class II) and B-cell epitopes of SARS-CoV were explored in the IEDB by querying “Severe acute respiratory syndrome-related coronavirus (taxonomy ID: 694009). We restricted our search to “Linear Epitope” and “Positive Assays Only” to include linear epitopes with at least one positive assay for T cell and B cell, respectively, while keeping all other parameters as default.

### RNAi-Based Therapeutics

#### Potential Small Interfering RNAs (siRNAs)

We used the VIRsiRNApred (Qureshi et al., 2013) and desiRm (Ahmed and Raghava, 2011) programs for the prediction of siRNAs against SARS-CoV-2. VIRsiRNApred is a virus-specific method, and we used model-2, constructed by employing different features like the hybrid nucleotide frequencies, binary pattern, and thermodynamic properties of 1725 viral siRNAs. Further, only highly efficacious siRNAs (inhibition more than or equal to 60%) were considered. Additionally, potential siRNAs (predicted efficacy score greater or equal to 1) were also identified using the desiRm tool. Moreover, the off-targets of the siRNAs were also predicted. Additionally, the immunomodulatory impact was also deduced by the imRNA tool, which explores the immunomodulatory and non-immunomodulatory potential of siRNAs (Nagpal et al., 2017).

#### Putative MicroRNAs (miRNAs)

Similarly, we have also identified miRNAs for SARS-CoV-2 using a two-step method. In the first step, the VMir algorithm was utilized to predict the precursor miRNA (pre-miRNA) hairpins using the default parameters (Sullivan and Grundhoff, 2007), while in the second step, mature miRNAs were identified using the Mature Bayes tool (Gkirtzou et al., 2010).

#### Single Guide RNAs (sgRNAs)

For the identification of all of the possible single guide RNAs (sgRNAs), we used the ge-CRISPR tool/pipeline (Kaur et al., 2016). Prediction of sgRNAs was performed based on the Protospacer Adjacent motif (PAM) for the SARS-CoV-2 genome. The underlying algorithm scans all the “NGG” motifs in the genome for both the forward and reverse strands and picks up putative sgRNAs 20 nucleotides upstream of the motifs found thereby. In the geCRISPR tool pipeline 2, ge-CRISPRr was selected, which employs a regression-based algorithm to predict sgRNA efficiency (0–100%).

#### Coronavirus (CoV) Primers

To obtain an exhaustive list of primers, two separate approaches were employed in the study. First, we searched for the experimental primers previously used for the detection of coronaviruses (CoVs). For this, a literature search was performed in PubMed using the different keywords “coronavirus” and “primers^∗^.” Overall, 185 papers were obtained (on 12/02/2020) and were further examined to collect the oligonucleotide primer information. Meta-information was collected for each primer pair, mainly primer name, sequence, orientation, start-end, genome name, gene name, strain, accession number, etc.

Furthermore, in the second approach, we designed primer pairs for SARS-CoV-2 based on different parameters using the PrimerDesign-M tool (Yoon and Leitner, 2015). We used the multiple fragment option with Flex design for fragment overlap. Further, the start and end of the target region were specified for the region of interest. Additionally, primer length range (20–25), detection limit (5%), complexity limit (2%, one degenerate position), window size (10-mer), and dimer ratio (0.9) were used. A 5°C difference between the melting temperatures (Tm) of the forward and reverse primer in pairs was set.

#### Glycosylation in CoVs

We also performed prediction and analysis of glycosylation sites (C, N, and O) for all of the proteins of SARS-CoV-2. Additionally, the other six CoVs, i.e., SARS, MERS, 229E, OC43, NL63, and HKU1, were also investigated for the identification of glycosylation sites. We used NetCGlyc*v*1.0 (Julenius, 2007), NetNGlyc*v*1.0 (Blom et al., 2004), and NetOGlyc *v*.4.0 (Steentoft et al., 2013) for C-linked, N-linked, and O-linked glycosylation, respectively. Additionally, we also compared the glycosylation sites in these seven CoVs to elucidate the conservation between them.

#### Phylogenetics

For the phylogenetic analysis, 48 representative coronavirus genomes and their corresponding proteomes (latest as of 17/02/2020) were selected, and their evolutionary relationship was identified using MEGA 10.1.7 (Kumar et al., 2018). Genome sequence alignment was performed using the MUSCLE (Edgar, 2004) algorithm integrated within the MEGA program. For both the genomes and the proteomes, the phylogenetic tree was constructed based on the maximum likelihood (ML) method. In the case of genomes, the ML tree was constructed following the general time-reversible (GTR) model using a discrete Gamma distribution (+G). Similarly, for proteomes, the LG (Le and Gascuel, 2008) model using discrete Gamma distribution (+G) was used for building the ML tree. The robustness of the tree topology was calculated using the bootstrap method (Felsenstein, 1985) with 1000 bootstrap replications for the genome-based tree, while the corresponding proteome tree was built using 100 bootstrap replicates.

#### Codon Usage and Nucleotide Composition

Complete nucleotide sequences of all coding regions of SARS-CoV-2 were retrieved from NCBI (NC_045512.2). To gain insight into the codon usage, different parameters such as the number of amino acids, number of codons, relative synonymous codon usage (RSCU), rare codons, and codon context were calculated using Anaconda software (Moura et al., 2005). The nucleotide composition (in percentages) of A, U, G, C, A + U, G + C, G + A, G + T, A + T, A + C, C + T, GC1, GC2, and GC3 of all coding regions was calculated using the online program CAIcal^14^ (Puigbo et al., 2008). Additionally, the estimation of codon adaptation of the SARS-CoV-2 in the host, the effective number of codons (ENc), and the Codon Adaptation Index (CAI) value were calculated using CAIcal software. In the analysis, the synonymous codon usage pattern of the viral host *(Homo sapiens)* was taken as the reference, and the CAI values of the coding regions of SARS-CoV-2 were calculated after comparison with the reference. The codon usage pattern of *Homo sapiens* was retrieved from the Codon Usage Database^15^.

#### Protein Structure Prediction, Comparison, and Analysis

In order to elucidate important aspects and structural conservation of SARS-CoV-2 proteins, *in silico* structure prediction and analysis was performed for six proteins of CoVs, namely, the four structural proteins, S, E, N, and M, and two non-structural proteins, RNA-dependent RNA polymerase (RdRp) and Helicase. The structures of the above-mentioned proteins from seven different CoVs were modeled using SWISS-MODEL (Waterhouse et al., 2018). Further, 3D structural comparison and analysis were also performed and represented using PyMOLv1.7.4^16^. All of the predicted structures of proteins for these seven CoVs, including all SARS-CoV-2 proteins, are also provided on our web resource with the visualization and download facility.

#### CoronaVR Resource Development

“CoronaVR” was built and hosted in the Linux environment on an Apache HTTP server (v2.2.17) utilizing the LAMP (Linux, Apache HTTP Server, MySQL, and PHP) open-source platform. The backend is mainly supported by MySQL for effective data management. The web-interface was created the employing PHP, HTML, CSS, and JavaScript. In-house scripts were also developed to process and perform data processing. Further, a Corona genome browser was also included.

## Results and Discussion

**FIGURE 2:**
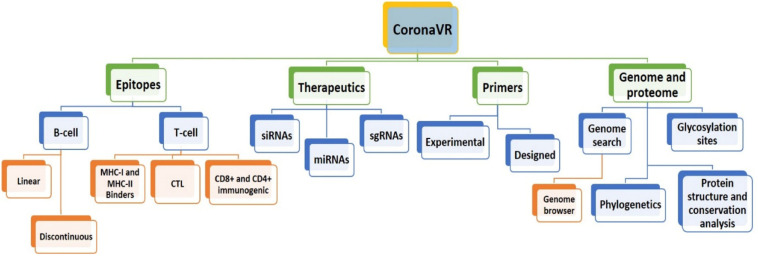
Architecture of the CoronaVR compendium.

We have developed an integrative resource equipped with a compendium of putative anti-CoV solutions and genomic knowledge to assist the scientific community in dealing with the deadly public health threat of COVID-19. For this, using a systematic and dedicated approach, we developed “CoronaVR.” The resource is well-organized into different sections for interactive navigation. It is broadly categorized into the separate divisions, viz., epitopes, therapeutics, primers, and genomes. It also comprises tools for analysis and visualization. A complete overview of the CoronaVR resource is illustrated in Figure 2.

### CoronaVR Genomes and Browser

We have compiled 365 complete genome sequences of human infective CoVs with sizes ranging between 27 and 32 kb. A catalog of CoVs is also provided in the resource in the genomes section. A categorywise advance search facility using different criteria, viz., geographic area (e.g., Asia), country (e.g., China), Year (2003, 2019, etc.), Length range, etc., is also implemented for sequence data retrieval. Detailed meta-information, like genome accession number, virus name, strain/isolate, length, geographical area, country of origin, etc., is provided. To navigate through the seven reference human-infecting CoVs, we have also developed a graphical genome browser backed by JBrowse. Different color codes depict distinct genome features with semantic navigation, a ruler, and zooming.

### Putative Vaccine Epitopes

We used the IEDB MHC-I binding prediction tool to predict MHC-I binders from protein sequences of SARS-CoV-2. The consensus method was used for binding prediction, and peptides with IC50 less than 50 nM were selected as strong binders. These predicted binders of each protein sequence are subjected to antigenicity, toxicity, and allergenicity prediction. Out of the total 424 non-allergenic, non-toxic, and antigenic MHC-I binders, 168 peptides were found to be 100% conserved within the SARS-CoV protein sequences (Supplementary Table S1). The number of peptide sequences that remained after each prediction step is shown in Supplementary Table S2.

Likewise, we used NetCTLpan *v*1.1 to predict CD8^+^ T-cell epitopes from protein sequences. Prediction was made on 12 HLA supertypes (A1, A2, A3, A24, A26, B7, B8, B27, B39, B44, B58, and B62) with the remaining parameters as default. The peptides with a% Rank less than 1% (<E) were selected as per the default selection criteria of the web server. Overall, 1499 CTL epitopes were predicted from 11 proteins of SARS-CoV-2. Out of 1499 predicted CTL epitopes, 765 were found to be antigenic. Further analysis of these 765 peptides showed that 754 were non-toxic and 273 were non-allergenic (Supplementary Tables S3, S4). These 273 non-allergenic CTL epitopes were analyzed for sequence conservancy and population coverage. Of the 273, 169 epitopes were found to be 100% conserved with SARS-CoV sequences, while the others were conserved to variable degrees (Supplementary Table S3). Potential CTL epitopes pertaining to the four structural proteins (E, S, M, and N) conserved in both SARS-CoV-2 and SARS-CoV are provided in Table 1.

**TABLE 1:** Potential CTL epitopes conserved in SARS-CoV-2 and SARS-CoV.

*Protein*	*Peptides*	*Start*	*Stop*
E	SVLLFLAFV	16	24
E	LLFLAFVVF	18	26
E	FLAFVVFLL	20	28
E	FLLVTLAIL	26	34
E	YVYSRVKNL	57	65
M	LWPVTLACF	57	65
M	FVLAAVYRI	65	73
M	SELVIGAVI	136	144
M	ATSRTLSYY	173	181
M	TSRTLSYYK	174	182
N	LSPRWYFYY	105	113
N	SPRWYFYYL	106	114
N	KTFPPTEPK	366	374
S	VRFPNITNL	331	339
S	YQPYRVVVL	512	520
S	PYRVVVLSF	514	522
S	LLFNKVTLA	832	841
S	WTFGAGAAL	898	906
S	FAMQMAYRF	910	918
S	AEIRASANL	1030	1038
S	VVFLHVTYV	1075	1083
S	KEIDRLNEV	1197	1205
S	VLKGVKLHY	1282	1290

*E, envelope, M, membrane, N, nucleocapsid, S, spike.*

Furthermore, immunogenic peptides restricted to MHC-I were identified using the “IEDB Class I Immunogenicity tool” with default parameters. We found 236 immunogenic epitopes in total, with envelope (E) and ORF8 having no predicted immunogenic MHC-I epitopes. Out of these 236 epitopes, only 33 were found to be antigenic according to the Vaxijen score and were selected for toxicity and allergenicity prediction. These 33 peptides were found to be non-toxic, while 21 were non-allergenic (Supplementary Table S5). The numbers of selected epitopes from each protein used for prediction at each step are shown in Supplementary Table S6. Finally, these 21 immunogenic and non-allergenic epitopes from different proteins were selected for conservancy analysis and population coverage (Table 2). The conservancy analysis showed that only two immunogenic CD8 + T-cell epitopes (present in ORF7b) were 100% conserved with the SARS-CoV sequences, while five were 90% conserved (Supplementary Table S5).

**TABLE 2:** Potential immunogenic CD8^+^ T-cell epitopes pertaining to SARS-CoV-2.

*Protein*	*Peptides*	*Start*	*End*
*M*	RINWITGGIA	72	81
*M*	VYRINWITGG	70	79
*NSP3*	DCEEEEFEPS	935	944
*NSP2*	EHEHEIAWYT	233	242
*NSP3*	GDCEEEEFEP	934	943
*2′-O-ribose methyltransferase (NSP16)*	GHFAWWTAF	6983	6991
*2′-O-ribose methyltransferase (NSP16)*	GHFAWWTAFV	6983	6992
*2′-O-ribose methyltransferase (NSP16)*	HFAWWTAFV	6984	6992
*NSP2*	KLNEEIAIIL	468	477
*NSP2*	LNEEIAIILA	469	478
*NSP4*	LVPFWITIA	3135	3143
*NSP4*	LVPFWITIAY	3135	3144
*2′-O-ribose methyltransferase (NSP16)*	MGHFAWWTA	6982	6990
*2′-O-ribose methyltransferase (NSP16)*	MGHFAWWTAF	6982	6991
*NSP4*	PLVPFWITIA	3134	3143
*NSP4*	TKHFYWFFS	3150	3158
*NSP4*	VPFWITIAY	3136	3144
*NSP4*	VPFWITIAYI	3136	3145
*S*	NVTWFHAIHV	61	70
*ORF7b*	IMLIIFWFSL	23	32
*ORF7b*	MLIIFWFSL	24	32

*E, envelope, M, membrane, N, nucleocapsid, S, spike, NSP, non-structural protein.*

Similarly, MHC-II binders from SARS-CoV-2 protein sequences were predicted using the IEDB MHC-II binding prediction method. As per IEDB recommendation, we used peptides with IC50 less than 50 nM as a cut-off to select strong binders (Wang et al., 2008). Using this selection criterion, we obtained 1478 strong binders restricted by MHC-II alleles. These predicted binders were subjected to antigenicity prediction. Out of the 1478 predicted MHC-II peptides, 831 were found to be antigenic on the basis of a Vaxijen score greater than 0.4. After subjecting these antigenic peptides prediction of Interferon gamma (IFN-γ) secreting peptides, only 304 were found to be positive according to the IFNepitope score. Toxicity prediction reduced this number to 296 (i.e., non-toxic). These 296 peptide sequences were then further subjected to allergenicity prediction, and 194 peptides were found to be non-allergenic, while 102 were allergenic. These 194 peptides can be used as vaccine candidates to elicit helper T-cells (CD4^+^) (Supplementary Tables S7, S8). Additionally, epitope conservancy and population coverage by these epitopes were also determined. Out of these 194 peptides, only three sequences were 100% conserved (S: 2, ORF1ab: 1) within the SARS-CoV sequence, while the total number of sequences with more than 90% conservancy was 78, with a variable degree of conservation with other CoVs (Supplementary Table S7).

Prediction of immunogenic CD4 + T-cell epitopes from SARS-CoV-2 proteins using the “CD4 T cell immunogenicity prediction tool” available at the IEDB resulted in 319 immunogenic peptides. Out of these 319 epitopes, 132 were found to be antigenic. Further testing of these peptides for toxicity resulted in 129 peptides where no “non-toxic” peptide was found in ORF10. Among these 129 peptides, 44 were found to be non-allergic (Supplementary Tables S9, S10) and, thus, can be safely used for vaccine formulations after testing them further for conservancy and population coverage. The conservancy analysis showed that 19 epitopes were 100% conserved with SARS-CoV sequences, while there were 28 sequences in total that were more than 90% conserved with SARS-CoV (Supplementary Table S9).

Likewise, 320 linear B-cell epitopes were predicted from the SARS-CoV-2 proteins. Predicted epitopes varied in length from 111 (maximum) to a single amino acid residue (minimum). Of these epitopes, only 135 were found to be antigenic using Vaxijen. Toxicity prediction of these 135 antigenic peptides resulted in 126 non-toxic and 9 toxic sequences. Allergenicity prediction of these non-toxic peptides showed that only 65 sequences were predicted to be non-allergenic, while the remaining 61 were allergenic. These epitope sequences and their lengths, start and end points in a protein, and conservancies are shown in Supplementary Table S11. Supplementary Table S12 shows the protein-wise distribution of the counts of these epitopes. These 65 sequences were further tested for conservancy with other coronavirus strains (Supplementary Table S11).

Of these 65 epitopes, 20 were found to be 100% conserved and 26 sequences (*N* = 2, *S* = 1, ORF1ab = 23) were more than 90% conserved with SARS-CoV (Table 3). These 20 sequences are located in ORF1ab polyprotein in various regions. One Spike glycoprotein (S) epitope (^404^GDEVRQIAPGQTGKIA DYNYKLP^426^) with a length of 23-mer was found to be 91.3% conserved with the SARS-CoV spike protein. For envelope protein, only one sequence (^57^YVYSRVKNLNSSRVP^71^) was conserved within SARS-CoV, with 80% conservancy. Nucleocapsid protein (N) had two sequences (^226^RLNQLESKMS GKGQQQQGQTVTKKSAAEASKKPRQKRTATKA^267^ and ^276^R RGPEQTQGNFGDQELIRQGTDYK^299^) that were more than 95% conserved with the SARS-CoV sequence (Supplementary Table S11).

**TABLE 3:** Putative linear B-cell epitopes for SARS-CoV-2 and SARS-CoV.

Protein	Peptides	Start	End	Length
N	RLNQLESKMSGKGQQQQGQTVTKKSAAEASKKPRQKRTATKA	226	267	42
N	RRGPEQTQGNFGDQELIRQGTDYK	276	299	24
S	GDEVRQIAPGQTGKIADYNYKLP	404	426	23
Leader protein (NSP1)	LPVLQV	18	23	6
NSP3	SYKDWSYSGQ	1510	1519	10
NSP3	FPDLNG	1960	1965	6
NSP3	TRQVVNV	2747	2753	7
3C-like proteinase (NSP5)	MAFPSGK	3269	3275	7
3C-like proteinase (NSP5)	YNYEPLTQDH	3500	3509	10
NSP7	VQSKMSD	3858	3864	7
NSP8/NSP9	KLQNNELSPVAL	4138	4149	12
RDRP (NSP12)	PCGTGTSTDV	4413	4422	10
RDRP (NSP12)	TFSNYQHEET	4468	4477	10
RDRP (NSP12)	VAFQTVKPGNFNKDFYDFAVSKGFFKEGSSVEL	4797	4829	33
RDRP (NSP12)	LKYAISAKNR	4936	4945	10
RDRP (NSP12)	KPGGTSSGDATT	5068	5079	12
RDRP (NSP12)	WTETDLTKGP	5192	5201	10
Helicase (NSP13)	TCVGSDNVTDFNAIATCDWTNAGDYILANTCTE	5420	5452	33
Helicase (NSP13)	FEKGDYG	5524	5530	7
Helicase (NSP13)	PAPRTLLTKGTLEPE	5730	5744	15
Helicase (NSP13)	LYDKLQ	5905	5910	6
Helicase/3′-5′ Exonuclease	RNVATLQAENVTG	5919	5931	13
3′-5′ Exonuclease (NSP14)	MYKGLPW	6078	6084	7
3′-5′ Exonuclease (NSP14)	GFTGNLQSNHDLYCQVHGNAHVA	6173	6195	23
2′-O- ribose methyltransferase (NSP16)	DKGVAP	6873	6878	6
2′-O- ribose methyltransferase (NSP16)	IQLSSYSLFDMSKFPLKLRG	7035	7054	20

The epitopes from ORF1ab polyprotein, with length greater than 9-mer, were found to be variously conserved within ORF1ab polyprotein from the other six viruses. Two sequences (KLQNNELSPVAL and SYKDWSYSGQ) each of length 12-mer and 10-mer were conserved within four other coronavirus strains, while few are conserved within only SARS-CoV. No epitope sequences were found to be conserved within these five coronavirus strains according to our set criteria (Supplementary Table S11).

Further, a total of 37 conformational B-cell epitopes were predicted using the Ellipro method (Supplementary Table S13). The top 10 sequences of these 37 predicted epitopes had protrusion scores lying between 0.77 and 0.99. A high protrusion index (PI) value means enhanced solvent availability. Among these 10 sequences, 5 sequences belonging to the proteins ORF3a [(M1, D2, L3, F4, M5, R6), (T9, I10, G11, T12, V13, T14, L15)], ORF7b (E39, T40, C41, H42, A43), ORF8 (E92, P93, K94), and ORF10 (R24, N25, Y26) had PI scores above 0.80. The two highest-scoring peptides belonged to ORF3a, with PI scores of 0.99 and 0.94, respectively (Supplementary Table S13).

A few recent immunological studies have experimentally validated several epitope sequences and found some to be positive in qualitative/quantitative assays against SARS-CoV-2 proteins, specifically spike glycoprotein (Poh et al., 2020; Smith et al., 2020; Yi et al., 2020; Yuan et al., 2020).

Poh et al. (2020) have found 2 linear B-cell epitopes (S14P5 and S21P2) in the spike protein of SARS-CoV-2 and our predicted linear B-cell epitope sequences, namely- ^555^**SNKKFLPF**^562^ and ^807^PD**PSKPSK**^814^, in the spike protein of SARS-CoV-2 mapped on these two experimentally validated epitopes. These epitopes were found to be antigenic and non-toxic but allergenic in nature.

A study published in Science by Yuan and group found a conformational B-cell epitope in the receptor-binding domain that was highly conserved between SARS-CoV-2 and SARS-CoV (Yuan et al., 2020). We have also predicted a linear B-cell epitope that is a part of this discontinuous epitope with the sequence ^369^**YNSASFSTFKCYGVSPTK**LND**L**CFT^393^. This epitope was found in our analysis to be antigenic, non-toxic, and non-allergenic, having 84% sequence conservancy with SARS-CoV.

Similarly, Yi et al. (2020) also found some key residues in the spike protein of SARS-CoV-2 that interact with ACE2 as well as with neutralizing antibodies. These residues mapped on our predicted linear B-cell epitope ^369^**YNSASFSTFKCYGVSPTK**LND**L**CFT^393^ (mentioned above).

Trevor et al., found nine MHC-I-restricted T-cell epitopes in the spike protein of SARS-CoV-2 (Smith et al., 2020). Out of these nine epitopes, three were found to match with our predicted epitopes. “**VLSFELLHA**” mapped on one of the epitopes (V**VLSFELLHA**PATVC) and was found to be antigenic, non-toxic, and non-allergenic in our analysis. In the same way, **“VVFLHVTYV”** was predicted to be positively antigenic, non-toxic, and non-allergenic and completely mapped on an epitope (PHG**VVFLHVTYV**PAQ) found in the above-mentioned study. These two sequences can be used as vaccine candidates to elicit the adaptive arm of the immune system and provide protection against SARS-CoV-2. Epitope sequence “KIAD**YNYKL**,” predicted as a positive epitope by this study, also had a few residues matching with an experimentally confirmed epitope **(YNYKL**PDDFTGCVIA). However, it was found to be allergenic in our study.

### Population Coverage Analysis

The T-cell epitopes selected following the conservancy analysis were used to compute population coverage. We used the population coverage tool offered by the IEDB (see text footnote 14) to compute the population covered by predicted MHC-I, MHC-II binders, and CTL epitopes from SARS-CoV-2 (Bui et al., 2006).

The maximum population coverage of predicted MHC-I binders (which are also antigenic, non-toxic, and non-allergenic) was found for the European population (97.71%), which was followed by North America, West Indies, West Africa, Southeast Asia, Northeast Asia, North Africa, Oceania, South Africa, South Asia, East Africa, South America, Southwest Asia, Central Africa, and Central America, with predicted population coverages (PPC) of 97.48, 96.96, 92.96, 92.68, 92.61, 92.46, 91.64, 88.81, 88.78, 86.85, 85.18, 84.32, 83.82, and 7.76%, respectively, as shown in Supplementary Table S14.

The highest PPC for CTL epitopes was also found for the European population (95.66%), which was immediately followed by the North American population (87.54%). The PPC for Northeast Asia, including China (the area of COVID-19 outbreak), covered by these epitopes was quite low (65.65%) as compared to the high PPC (92.61%) for MHC-I binders for the same region. For MHC-II binders, the highest PPC was observed for the North American population (99.99%) closely followed by the European population (99.92%). Here, the area of Northeast Asia also had a high estimated PPC (93.81%). It is to be noted that the estimated PPC for European countries including Italy (most effected by COVID-19 along with China, United States, and Spain to date) provided by our predicted epitopes was very high (>99%) (Supplementary Table S14).

### T-Cell and B-Cell Epitopes of All Coronaviruses

The search for T-cell and B-cell epitopes from all global coronaviruses was performed in the IEDB, which harbored details for the following coronaviruses: Alphacoronavirus 1, Avian coronavirus, Betacoronavirus 1, Coronavirus HKU15, HCoV-229E, MERS-CoV, Murine coronavirus, Porcine epidemic diarrhea virus, SARS-CoV, and Swine acute diarrhea syndrome-related coronavirus. We obtained 320 positive T-cell epitopes, with 778 T-cell assays related to these epitopes. Similarly, 663 positive B-cell epitopes with 1568 B-cell assays were found in IEDB. Of these 663 epitopes, 582 were linear and 81 were conformational. The conservancy analysis of T-cell and B-cell epitopes from these coronaviruses with SARS-CoV-2 proteins showed that 41 unique T-cell and 83 linear B-cell epitopes were 100% conserved within SARS-CoV-2. Only Humans (*Homo sapiens*) and various experimental mice (*Mus musculus*) were found as hosts for these coronaviruses in the case of T-cell epitopes. However, in the case of linear B-cell epitopes from other coronaviruses that shared 100% conservancy with SARS-CoV-2, various animals such as the Formosan rock macaque (*Macaca cyclopis*), Guinea pig (*Cavia porcellus*), and Rabbit (*Oryctolagus cuniculus*) were also observed as hosts as well as *Homo sapiens* and *Mus musculus*.

### Cross-Protective Epitopes (CPEs) Between SARS-CoV and SARS-CoV-2

At a time when a vaccine is urgently required against SARS-CoV-2, the non-availability of epitope information for it is a shortcoming that may lengthen the vaccine development process. To help the researchers in developing a SARS-CoV-2 vaccine, we sought to identify the cross-protective epitopes (CPEs) and unique epitopes (UE) based on antigenic similarities and differences between SARS-CoV epitopes and SARS-CoV-2 protein sequences. For this, we extracted 119 T-cell and 405 linear B-cell epitope sequences of SARS-CoV (ID: 694009) available on the IEDB. These 119 T-cell epitopes were dispersed in 51 and 68 MHC class I and class II alleles, respectively. The conservancy analysis to find cross-protective epitopes was performed by mapping SARS-CoV T- and B-cell epitopes on SARS-CoV-2 proteins. Of the 119 T-cell epitopes, 27 potential cross-protective epitopes were found with 100% conservancy (no mutation) distributed in four different proteins (N: 13, S: 12, ORF1ab: 1, and M: 1) of SARS-CoV-2. Altogether, 75 T-cell epitopes of SARS-CoV were found with high sequence identity (>80%) with SARS-CoV-2. On the other hand, 13 sequences had moderate similarity (>70% but <80%) and 29 had low similarity (<70%) and can be considered as unique in SARS-CoV-2 (Supplementary Table S15).

We also checked the IFNepitope score (for MHC-II epitopes), toxicity, and allergenicity of these experimentally confirmed T-cell epitopes of SARS-CoV and their corresponding sequences in SARS-CoV-2. Among the 27 epitopes that were 100% conserved, 14 were predicted to be non-toxic and non-allergenic (Table 4). Out of the 7 MHC-II restricted T-cell epitopes (100% conserved), only 2 were found to have positive IFNepitope scores, and only one (^306^AQFAPSASAFFGMSR^320^) was found to be non-toxic and non-allergenic. Supplementary Table S15 lists T-cell epitopes of SARS-CoV with conservancy with SARS-CoV-2 that fulfill other criteria [IFNepitope (for MHC II), Non-Toxic, Non-Allergenic] that can be used as vaccine candidates to provide cross-protection against each other.

**TABLE 4:** Potential cross-protective T-cell epitopes (vaccine candidates) against SARS-CoV-2 and SARS-CoV.

Protein	MHC type	SARS-CoV-2 Epitopes	Start	End	Length	Allergenicity
S	MHC-I	VNFNFNGL	539	546	8	Allergen
N	MHC-I	ILLNKHID	351	358	8	Allergen
S	MHC-I	ALNTLVKQL	958	966	9	Non-Allergen
N	MHC-I	ALNTPKDHI	138	146	9	Allergen
S	MHC-I	FIAGLIAIV	1220	1228	9	Non-Allergen
N	MHC-I	GMSRIGMEV	316	324	9	Non-Allergen
N	MHC-I	ILLNKHIDA	351	359	9	Allergen
N	MHC-I	LALLLLDRL	219	227	9	Non-Allergen
S	MHC-I	LITGRLQSL	996	1004	9	Allergen
N	MHC-I	LLLDRLNQL	222	230	9	Allergen
N	MHC-I	LQLPQGTTL	159	167	9	Allergen
S	MHC-I	NLNESLIDL	1192	1200	9	Allergen
S	MHC-I	RLNEVAKNL	1185	1193	9	Allergen
ORF1ab	MHC-I	VLAWLYAAV	3467	3475	9	Non-Allergen
S	MHC-I	VLNDILSRL	976	984	9	Non-Allergen
S	MHC-I	VVFLHVTYV	1060	1068	9	Non-Allergen
M	MHC-I	TLACFVLAAV	61	70	10	Non-Allergen
N	MHC-I	MEVTPSGTWL	322	331	10	Non-Allergen
N	MHC-I	RRPQGLPNNTASWFT	40	54	15	Allergen
N	MHC-II	AQFAPSASAFFGMSR	305	319	15	Non-Allergen
N	MHC-II	SPRWYFYYLGTGPE	105	119	15	Non-Allergen
N	MHC-II	VILLNKHIDAYKTFP	350	364	15	Allergen
S	MHC-II	GAALQIPFAMQMAYRF	891	906	16	Non-Allergen
S	MHC-II	MAYRFNGIGVTQNVLY	902	917	16	Non-Allergen
S	MHC-II	QALNTLVKQLSSNFGAI	957	973	17	Non-Allergen
N	MHC-I	LLNKHIDAYKTFPPTEPK	352	369	18	Allergen
S	MHC-II	QLIRAAEIRASANLAATK	1011	1028	18	Allergen

In parallel, the mapping of linear B-cell epitopes of SARS-CoV showed that out of 405 epitopes, 83 were 100% conserved in SARS-CoV-2 proteins (E: 1, ORF1ab: 1, M: 6, N: 32, and S: 43). Comprehensively, there were 237 epitopes with sequence identity >80% (Supplementary Table S16). Toxicity and allergenicity prediction of SARS-CoV epitopes resulted in 45 non-toxic and non-allergenic sequences that were 100% conserved with SARS-CoV-2 proteins. These 45 shared epitopes between SARS-CoV and SARS-CoV-2 can provide cross-protection against each other and can be utilized as potent linear B-cell epitopes to elicit humoral immunity (Table 5).

**TABLE 5:** Potent cross-protective B-cell epitopes (vaccine candidates) against SARS-CoV-2 and SARS-CoV.

Protein	SARS-CoV-2 Epitopes	Start	End	Length
E	RVKN	61	64	4
M	LEQWNLVIGFLFL	17	29	13
M	PKEITVATSRTLSYYKL	165	181	17
M	GRCDIKDLPKEITVATSR	157	174	18
N	GSFCTQLN	278	285	8
N	LPQRQKKQ	382	389	8
N	SQASSRSS	180	187	8
N	TFPPTEPK	362	369	8
N	LPQGTTLPKG	161	170	11
N	GFYAEGSRGGSQASS	170	184	15
N	GSRGGSQASSRSSSR	175	189	15
N	KTFPPTEPKKDKKKK	361	375	15
N	TTLPKGFYAEGSRGG	165	179	15
N	YKTFPPTEPKKDKKK	360	374	15
N	FFGMSRIGMEVTPSGTW	314	330	17
N	KHWPQIAQFAPSASAFF	299	315	17
N	QFAPSASAFFGMSRIGM	306	322	17
N	PKGFYAEGSRGGSQASSR	168	185	18
N	QLPQGTTLPKGFYAEGSR	160	177	18
N	KHIDAYKTFPPTEPKKDKKK	355	374	20
N	VTQAFGRRGPEQTQGNFGDQ	270	289	20
N	QLPQGTTLPKGFYAEGSRGGSQ	160	181	22
S	AMQMAYRF	899	906	8
S	GAGICASY	667	674	8
S	KGIYQTSN	310	317	8
S	DDSEPVLKGVKLHYT	1259	1273	15
S	DKYFKNHTSPDVDLGD	1153	1168	16
S	AISSVLNDILSRLDKVE	972	988	17
S	EAEVQIDRLITGRLQSL	988	1004	17
S	EELDKYFKNHTSPDVDL	1150	1166	17
S	GAALQIPFAMQMAYRFN	891	907	17
S	IRQGTDYKHWPQIAQFA	292	308	17
S	KEIDRLNEVAKNLNESL	1181	1197	17
S	MAYRFNGIGVTQNVLYE	902	918	17
S	PELDSFKEELDKYFKNH	1143	1159	17
S	PFAMQMAYRFNGIGVTQ	897	913	17
S	QALNTLVKQLSSNFGAI	957	973	17
S	RLITGRLQSLQTYVTQQ	995	1011	17
S	SLQTYVTQQLIRAAEIR	1003	1019	17
S	TVYDPLQPELDSFKEEL	1136	1152	17
S	CKFDEDDSEPVLKGVKLHYT	1254	1273	20
S	EIDRLNEVAKNLNESLIDLQELGKYEQY	1182	1209	28
S	EIDRLNEVAKNLNESLIDLQELGKYEQY	1182	1209	29
S	DSFKEELDKYFKNHTSPDVDLGD ISGINASVV	1146	1177	32
S	ISGINASVVNIQKEIDRLNEVAK NLNESLIDLQELGKYEQYI	1169	1210	42

Overall, an immuno-informatics-driven methodology was implemented to discover the B-cell (linear and conformational) and T-cell (CD8^+^ and CD4^+^) epitopes, which can help researchers at the initial stage of the design of vaccine against SARS-CoV-2. With no experimentally confirmed epitopes of SARS-CoV-2 to date, we sought to address potential epitopes using various computational tools. We also considered various other properties, neglecting which may destroy the purpose of the development of a vaccine against SARS-CoV-2, such as antigenicity, toxicity, and allergenicity.

Some parallel studies are available that have identified different epitope components of SARS-CoV-2 through bioinformatics predictions (Ahmed et al., 2020; Baruah and Bose, 2020; Grifoni et al., 2020; Lucchese, 2020; Qiu et al., 2020). Grifoni et al., mapped experimentally confirmed epitopes of SARS-CoV on SARS-CoV-2 and predicted new epitope sequences as well (Grifoni et al., 2020). Ahmed et al., also mapped experimentally confirmed epitopes of SARS-CoV on SARS-CoV-2 and analyzed the population coverage for T-cell epitopes to find epitopes for vaccine formulation (Ahmed et al., 2020). Qui T. et al., searched for cross-protective epitopes on Spike protein of SARS-CoV-2 based on similarity with epitopes of SARS-CoV (Qiu et al., 2020). Lucchese et al., addressed pentapeptides of SARS-CoV-2 proteins absent in human as vaccine candidates (Lucchese, 2020). Bose et al., identified T- and B-cell epitopes in spike protein of SARS-CoV-2 using an immunoinformatics method (Baruah and Bose, 2020).

Our work is different from other studies in several aspects and gives various new insights that are important for designing vaccine formulations against SARS-CoV-2. The most important difference is that we are providing a unifying online platform for easy, free, and direct access to components to assist researchers. We have not limited our study to a selected few but have performed a comprehensive analysis of all proteins of SARS-CoV-2, specifically the structural proteins, to find vaccine candidates.

We performed antigenicity, toxicity, and allergenicity prediction of our addressed epitope sets since these are important considerations in vaccine formulation. For the MHC-II-restricted epitopes predicted in our study, we have also predicted the IFN-gamma-inducing ability of these peptides. We have performed cross-conservancy analysis of predicted epitopes with other coronaviruses causing diseases in Humans. To the best of our knowledge, no study has performed these analyses on SARS-CoV-2 proteins.

Our study suggests several epitopes as probable vaccine candidates on the basis of antigenicity, toxicity, and allergenicity along with IFN-gamma-inducing properties for MHC-II-restricted epitopes. The epitope mapping on the proteins of other human-infecting coronavirus strains showed conservancy to SARS-CoV to variable degrees. We found 169, 2, 19, and 20 CTL, immunogenic CD8^+^, immunogenic CD4^+^, and B-cell epitopes, respectively, with 100% sequence conservancy within SARS-CoV, which can be used as potent vaccine candidates against both of the viruses. However, very few sequences were found to be conserved with the other five coronaviruses, highlighting the fact that SARS-CoV-2 is quite different from these human-infecting viruses. This finely selected list of predicted epitopes of SARS-CoV-2 can be tested in future studies for the elicitation of immune response for their use as vaccine candidates. We have predicted T-cell epitopes in order to cover the Chinese ethnicity as well as the majority of the population around the world.

Several studies have highlighted the importance of the adaptive arm of the immune system (i.e., T-cells and B-cells) in providing protection against SARS-CoV (Wang et al., 2004; Xu and Gao, 2004; Chen et al., 2010; Channappanavar et al., 2014; Liu et al., 2017). We have identified SARS-CoV T-cell and B-cell epitopes with 100% conservancy in SARS-COV-2 proteins. These are cross-protective and can be used for designing a vaccine against SARS-CoV-2. A total of 27 T-cell epitopes of SARS-CoV were found that were fully conserved in different proteins (N: 13, S: 12, ORF1ab: 1, and M: 1) of SARS-CoV-2. We also checked peptide sequences of SARS-CoV-2 proteins that were found with variable levels of conservancy with SARS-CoV epitopes and predicted their antigenicity, toxicity, IFN-gamma-secreting ability, and allergenicity. We found one MHC-II-restricted epitope, namely, ^306^AQFAPSASAFFGMSR^320^, present in nucleocapsid of SAR-CoV that was 100% conserved within SARS-CoV-2 and was predicted to be antigenic, non-toxic, IFN-gamma-inducing and non-allergenic. Hence, this epitope sequence can be incorporated in designing a vaccine to provide cross-protection against SARS-CoV and SARS-CoV-2. We also found 45 shared linear B-cell epitopes between SARS-CoV and SARS-CoV-2 that were antigenic, non-toxic, and non-allergenic that can provide cross-protection against each other and can be utilized as potent vaccine candidates to elicit humoral immunity.

We expect that this study may help researchers in developing an inexpensive epitope-based vaccine against SARS-CoV-2 that may provide immunity to the entire world’s population.

### siRNAs and miRNAs

RNA interference-based silencing of viral genes provides an excellent alternative therapeutic tool. For this, we also explored and provided a compilation of putative efficient siRNAs against all of the genes of SARS-CoV-2. In total, 166 potent siRNAs with more than 60% inhibition were identified using the VIRsiRNApred algorithm. The different siRNAs targeting different genes of SARS-CoV-2 are provided in Supplementary Table S17. Correspondingly, 1163 putative siRNAs with efficacy scores equal to or more than 1 were also recognized utilizing the desiRm method and are provided on the server. For all of the siRNAs, the sense–antisense sequence, gene target, start-end, efficacy scores, immunomodulatory potential, and off-target information are provided on the CoronaVR resource. Additionally, we have also identified SARS-CoV-2 pre-miRNAs and mature miRNAs. Overall, 50 pre-miRNAs were identified, with a pair of mature miRNAs (5p and 3p). Complete information on precursor (hairpin) sequence, precursor length, location (start-end), genomic region, mature-miRNA sequence, GC content, etc., is provided (Supplementary Table S18).

### sgRNA-Based Genome Editing

Based on our analysis, 64 putative efficient sgRNAs were identified for SARS-CoV-2. Complete information like sgRNA sequences (5′-3′), PAM, start and end positions of the sgRNAs in the genome, GC%, and predicted sgRNA efficiency (%) is displayed in tabular format. This analysis will certainly help the scientific community to identify potential CRISPR targets and to design efficient sgRNAs against SARS-CoV-2 prior to experimental procedures. Highly efficient sgRNAs targeting SARS-CoV-2 are provided in Supplementary Table S19.

### Molecular Diagnostic Primers

The literature was searched in PubMed using different keywords, i.e., “coronavirus,” “homo-sapiens/humans,” and “primers^∗^,” and a total of 185 papers were retrieved. Overall, 198 primer sets specific for different strains of CoVs were obtained. Of these, 7 primer pairs are specific for SARS-CoV-2, 47 are for SARS-CoV, 25 are for MERS-CoV, and 107 are for the different HCoVs (229E-45, OC43-28, NL63-23, and HKU1-9). Additionally, we also identified three universal primer pairs, 6 sets of primers for beta-CoVs (SARS-CoV, MERS-CoV, OC43, and HKU1), and 2 primer sets specific to the alpha-CoVs (229E and NL63). These primers are specific for the particular genes, and some are applicable for the whole genome of the CoVs. Among all of them, 67 primers belong to the N gene, 6 primers are for gene E, 14 belong to the S gene, 9 primers are for gene M, 27 primers are specific for RdRp, 3 primer sets are for the UTR region, 17 are for ORF1a, 13 belong to ORF1b, 13 are for orf1ab, and 1 primer set is for ORF8.

Furthermore, we also designed specific primers for the different genes of SARS-CoV-2 using the Primer Design-M tool. In total, 21 primer sets were designed that are specific to the individual genes. Among these primer pairs for each gene, i.e., M, N, ORF3a, ORF6, ORF7a, ORF7b, and ORF10, 4 belong to ORF8 and 3 primer sets were designed for the S gene.

### Glycosylation in SARS-CoV-2

We also explored glycosylation sites in SARS-CoV-2. For this, three types of glycosylation sites, namely, C-linked, N-linked, and O-linked, were deduced. In total, 130 sites, i.e., 52 N-glycosylated (N-Gly) sites, and 78 O-glycosylated (O-Gly) sites were predicted. However, we could not find any C-mannosylated sites in SARS-CoV-2.

The protein-wise N-linked glycosylation sites are as follows M (1), E (2), S (17), ORF6 (1), ORF7b (1), ORF8 (1), N (2), nsp2 (3), Papain-like proteinase (8), Proteinase 3CL-PRO (2), nsp6 (1), nsp9 (1), nsp10 (2), Helicase (3), Guanine-N7 methyltransferase (3), Uridylate-specific endoribonuclease (2), and 2′-O-methyltransferase (2). In contrast, there are no single N-glycosylation sites found in ORF3a, ORF7a, ORF10, Host translation inhibitor nsp1, nsp4, nsp7, nsp8, and RdRp. Likewise, protein-wise O-linked glycosylation sites are as follows: S (3) and N (47), Host translation inhibitor nsp1 (1), Papain-like proteinase (14), nsp9 (1), RdRp (2), Helicase (6), Guanine-N7 methyltransferase (3), and Uridylate-specific endoribonuclease (1). The remaining proteins, viz., ORF3a, E, M, ORF6, ORF7a, ORF7b, ORF8, ORF10, nsp2, nsp4, Proteinase 3CL-PRO, nsp6, nsp7, nsp8, nsp10, and 2′-O-methyltransferase, do not contain any O-linked glycosylation sites.

### Phylogenomics

The 48 viral genomes and their corresponding proteome that were selected for the construction of the phylogenetic tree included 36 SARS-CoV-2 strains, and the remaining 12 were from SARS coronavirus, MERS, and different HCoVs strains, viz., NL63, HKU1, 229E, and OC43. A similar pattern of positioning of the viral taxa has been observed in the previous reports (Benvenuto et al., 2020a, b; Malik et al., 2020), where all of the SARS-CoV-2 strains were clustered together, indicating their uniqueness and identity when compared with the previously reported strains (Supplementary Figures S1, S2).

### Codon Usage and Bias Analysis

We analyzed the nucleotide composition, amino acid numbers, number of codons, relative synonymous codon usage (RSCU), rare codons, codon context, effective number of codons (ENC), and codon adaptation index (CAI) for the different genes of SARS-CoV-2. The nucleotide composition of all of the coding regions in SARS-CoV-2 revealed that the most and least frequent bases are T and G, respectively. The nucleotide frequencies were T > A > C > G (Supplementary Table S20). Also, the same frequency is observed for nucleotides at the third position (NT3s) of a codon. This shows that AT% > GC% in the SARS-CoV-2 genome (Supplementary Table S20). Further, codon numbers and RSCU were analyzed. This gives the ratio of expected to observed frequencies of synonymous codon usage by amino acids. An RSCU value of 1 indicates no bias in codon usage, whereas RSCU values <1 or >1 indicate negative and positive codon usage bias (Sheikh et al., 2020). From the RSCU values for different coding regions, the most preferred (RSCU ≥ 1.5) and the least favored codons (RSCU ≤ 0.5) are identified in Supplementary Table S21. A list of codons and RSCUs values for each coding region, i.e., ORF1ab, ORF1a, ORF3a, ORF6, ORF7a, ORF7b, ORF8, ORF10, E, M, N, and S are provided on the web resource. The analysis showed that U3s and A3s were the most recurrent nucleotides in the represented (preferred) codons and that C3s and G3s were the least frequent in all coding regions. Furthermore, gene-wise rare codons are also shown in a histogram. Simultaneously, gene-wise codon context analysis is also performed using Anaconda software, which provides the association between two codons, and the color scale indicates the preferred (green color with residual value more than +3), rejected (red color with residual value more than −3), and codon context with no bias (black color with residual values −3 to +3) codon pairs. The codon context for all of the coding regions is also provided on the server.

Moreover, codon usage bias is also deduced by determining the effective number of codons (ENC values) for different coding regions (Supplementary Table S22). ENC values range between 20 and 61. The higher ENC values indicate low codon bias, which indicates that more synonymous codons are used for amino acids (Chen et al., 2017). ENC values for different regions except ORF7b are greater than 40, which also shows low codon usage bias in SARS-CoV-2. In order to look into the relative adaptiveness of SARS-CoV-2 to its host, the codon adaptation index (CAI) was also calculated (Supplementary Table S22). CAI values range from 0 to 1, where 1 indicates that the gene always uses the most frequently used synonymous codons in the reference set (Castells et al., 2017). The mean CAI value for all coding regions is 0.686, which is greater than 0.5 and indicates moderate adaptability of SARS-CoV-2 to its host.

We have assessed all of the coding regions of SARS-CoV-2 for codon usage patterns, bias, and adaptability to the host. SARS-CoV-2 showed low GC content, like other members of the *Coronaviridae* family, such as SARS-CoV (Zhao et al., 2008), MERS-CoV (Chen et al., 2017), and BCoV (Castells et al., 2017). The RSCU values for each coding region in SARS-CoV-2 showed that almost all preferred codons ended with Us and As at the 3rd position of synonymous codons, whereas the least preferred ended with Gs and Cs at the 3rd position of synonymous codons. This showed that codon usage bias exists. The mean ENC value (46.845) of all coding regions in SARS-CoV-2 is greater than 40, which indicates low codon usage bias. This is consistent with previous studies on other SARS viruses like BCoV (mean ENC = 43.78), SARS-CoV (ENC = 48.99), Avian coronavirus Infectious bronchitis virus (ENC = 42.79), and Porcine epidemic diarrhea virus (ENC = 47.91) (Castells et al., 2017). The low codon usage bias indicates that SARS-CoV-2 might be able to use many synonymous codons to code for a single amino acid, which can be helpful in better survival and adaptability of a virus to its host. Further, to gain insight into the adaptation, the codon adaptation index (CAI) for each coding region was calculated in relation to the codon usage of its host, i.e., *Homo sapiens*. The mean CAI value of 0.686 showed better adaptability of SARS-CoV-2 to its host, *Homo sapiens*.

### Structural Analysis and Interpretation of SARS-CoV-2 Proteins

In this analysis, six different important proteins of SARS-CoV-2, i.e., RNA-dependent RNA polymerase (RdRp), Helicase, Spike (S), Envelope (E), Nucleocapsid (N), and membrane (M) were structurally analyzed and compared against the other human-infecting CoVs, namely, SARS-CoV, MERS-CoV and other HCoVs (OC43, 229E, NL63, and HKU1). The structures of these proteins from different CoVs along with all of the SARS-CoV-2 proteins were predicted (Supplementary Tables S23, S24). The templates used for the structure prediction are also provided. A structural comparison of these proteins is shown in Supplementary Figure S3. Additionally, root mean square deviation (RMSD) values for all of the protein comparisons are provided in Supplementary Table S25. Among these proteins, we have mainly focused on the two vital drug targets, viz., RdRp and S proteins.

### RNA-Dependent RNA Polymerase (RdRp)

**FIGURE 3:**
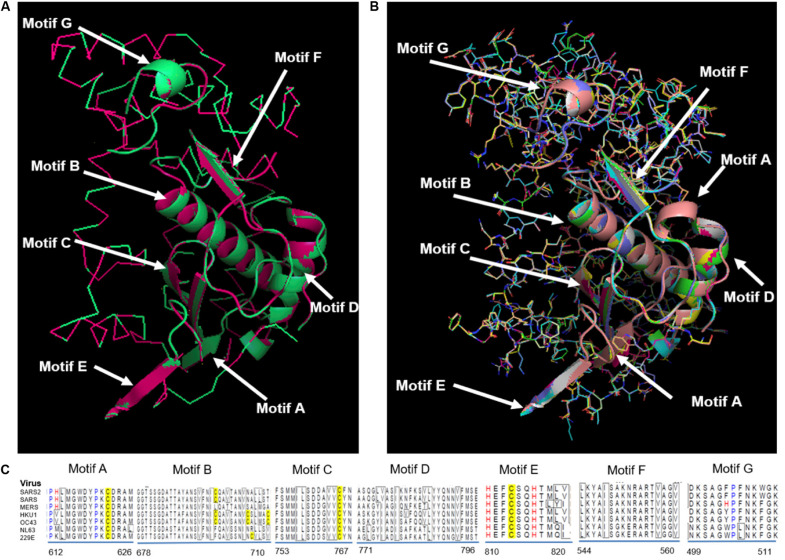
Structural comparison of RdRp. **(A)** SARS-CoV-2 RdRp with SARS-CoV RdRp, **(B)** SARS-CoV-2 compared with SARS-CoV, MERS-CoV, HKU1, OC43, NL63, and 229E. Different functional domains (A-G) of RdRp marked on structure, **(C)** Alignment showing conservation and variation among different motifs of RdRp from distinct CoVs.

RdRp proteins of SARS-CoV-2 and SARS-CoV share a remarkable 96.4% sequence identity, and other strains of CoVs, i.e., MERS, HKU1, OC43, NL63, and 229E and share 71, 67, 66, 59, and 58%, respectively (Supplementary Figure S4). RdRp involves a very large and deep groove as an active site for the polymerization of RNA (Supplementary Figure S3). Higher sequence conservation between RdRp enzymes makes it very likely that any potent agents developed for SARS-CoV and other strains of CoV RdRp will exhibit equally good potency and efficacy against SARS-CoV-2 RdRp. Further, Figure 3 shows a protein structure comparison of the RdRp of SARS-CoV-2 with SARS (Figure 3A) and seven different strains (Figure 3B) of coronavirus along with depictions of functional domains (A-G). Figure 3C shows the conservation and variation among different RdRp motifs of CoVs. SARS-CoV shows higher structural similarity with SARS-CoV-2 with a lower RMSD (Root Mean Square Deviation) value (0.005), while OC43 shows the highest divergence, with a RMSD of 0.122 (Supplementary Table S25).

### Membrane (M) Protein

Membrane (M) proteins represent the major protein component of the viral envelope. During viral assembly, M proteins play a very essential role by interacting with all of the other structural proteins. Its length ranges from 217 to 270 amino acid residues in most CoVs (Perrier et al., 2019). M proteins of SARS-CoV-2 and SARS-CoV share a remarkable 90% sequence identity, and other strains of CoVs, i.e., MERS, HKU1, OC43, NL63, and 229E share 42, 36, 40, 31, and 30%, respectively (Supplementary Figure S5). M protein contains three membrane-spanning hydrophobic segments, a small N-terminal domain situated outside the virion, and a large C-terminal domain that makes up half of the protein inside the virion. M proteins of some alphacoronaviruses contain an additional hydrophobic segment that functions as a signal peptide (de Haan and Rottier, 2005).

### Envelope (E) Protein

Envelope (E) protein of coronavirus is a small, integral membrane protein containing 76 to 109 amino acids that are involved in assembly, budding, envelope formation, and pathogenesis in the virus life cycle. The E proteins of SARS-CoV-2 and SARS-CoV share a remarkable 94% sequence identity, and other strains of coronavirus MERS, HKU1, OC43, NL63, and 229E share 36, 31, 31, 18, and 27%, respectively (Supplementary Figure S6).

### Helicase Protein

The unwinding of the double-stranded oligonucleotides into the single-stranded form using ATP during the replication cycle of the coronavirus is carried out by the enzyme helicase. Helicase proteins of SARS-CoV-2 and SARS-CoV share 99.83% sequence identity, and other strains of CoVs, i.e., MERS, HKU1, OC43, NL63, and 229E, share 72, 65, 68, 61, and 60%, respectively (Supplementary Figure S7). Structural conservation of these helicase proteins from different CoVs is also shown in Supplementary Figure S3. Helicase carries out the unwinding of nucleic acids during replication, recombination and DNA repair and is also involved in other biological processes, like movement of Holliday junctions, chromatin remodeling, displacement of proteins from nucleic acid, catalysis of nucleic acid conformational changes, and several aspects of RNA metabolism and mitochondrial gene expression (Adedeji and Lazarus, 2016). As the helicases of different coronaviruses are very homologous, helicase inhibitors are good and reliable anti-CoV treatment options. The helicase inhibitors can be categorized into two groups depending on their mechanism of action. Bananins and 5-hydroxychromone derivatives come under the first class of inhibitors, which inhibit viral replication *in vitro* by preventing the unwinding and ATPase activity of SARS-CoV helicase (Tanner et al., 2005; Kim et al., 2011). The second class of inhibitors includes those inhibitors that inhibit the unwinding but not the ATPase activity of helicase of CoV (Zumla et al., 2016).

### Nucleocapsid (N) Protein

This is a protein with numerous activities. Packaging of the viral genome into a helical ribonucleocapsid (RNP) is done by the nucleocapsid phosphoprotein. It plays a fundamental role during viral self-assembly. The suppression of RNA silencing and RNA interference that is triggered by either short hairpin RNAs or siRNAs is done by the N protein. The SARS-CoV-2 N protein is a phosphoprotein of 419 amino acids, sharing 90% sequence identity with the N protein of SARS-CoV. It shows a sequence identity of 38, 36, 48, 38, and 28% with the 229E, HKU1, MERS, OC43, and NL63 strains, respectively (Supplementary Figures S3, S8). N protein consists of two separate domains, an N-terminal domain (NTD) and a C-terminal domain (CTD), which are capable of binding to RNA *in vitro* via different mechanisms (Chang et al., 2006; Hurst et al., 2009). It also binds to nsp3 and M protein, nsp3 being the key component of the replicase complex (Sturman et al., 1980; Hurst et al., 2009, 2013). These protein interactions are likely to help in the packaging of the encapsulated genome into viral particles (Fehr and Perlman, 2015). Previous studies also show that N protein has been widely used as a diagnostic target of SARS-CoV. Viral N protein is considered to be a genetically stable protein, which is a primary criterion for selecting an efficient drug target candidate. It is even a therapeutic target in antiviral therapy due to its role in pathogenicity inside the cell (Chang et al., 2014).

### Spike (S) Glycoprotein

Surface glycoprotein or Spike (S) is a major immunogenic antigen of CoVs that is essential for interactions between a virus and host cell receptor, i.e., angiotensin-converting enzyme 2 (ACE2) and on S protein priming by a cellular protease, i.e., TMPRSS2 (Hoffmann et al., 2020). It has also been found that both SARS-CoV-2 and SARS-CoV use a common receptor, ACE2, for entry, and this is important for understanding the transmissibility and pathogenesis of SARS-CoV-2 (Hoffmann et al., 2020). Further, it is also estimated that SARS-CoV-2 S protein may have high binding affinity toward Human ACE2 (Smith et al., 2020; Zhang H. et al., 2020). In order to activate membrane fusion, virus entry, and syncytium formation, cleavage at the S1-S2 junction is necessary, and it undergoes structural rearrangement (Chan et al., 2015). When receptor-binding domain (RBD) of S1 subunit attaches to the host cell receptors, it causes conformational changes in the S2 subunit, which ultimately leads to the fusion of the viral and the cell membrane by bringing them into close proximity (Lu et al., 2014; Wrapp et al., 2020). Spike glycoprotein can be an ideal target for vaccine and antiviral development due to its role in receptor binding and membrane fusion. Various previous studies summarize the development of SARS vaccines based on the spike protein (Casais et al., 2003; Jiang et al., 2005; Roper and Rehm, 2009; Du et al., 2010). Various ideas and strategies (live-attenuated SARS-CoV, killed SARS-CoV, DNA vaccines, and viral-vectored vaccines) that have been used to develop vaccines against animal-CoVs could be used to develop SARS-CoV-2 vaccines as well. Additionally, a TMPRSS2 inhibitor may block the entry of the virus and might constitute a treatment option (Casais et al., 2003; Jiang et al., 2005; Roper and Rehm, 2009; Du et al., 2010; Morse et al., 2020).

**FIGURE 4:**
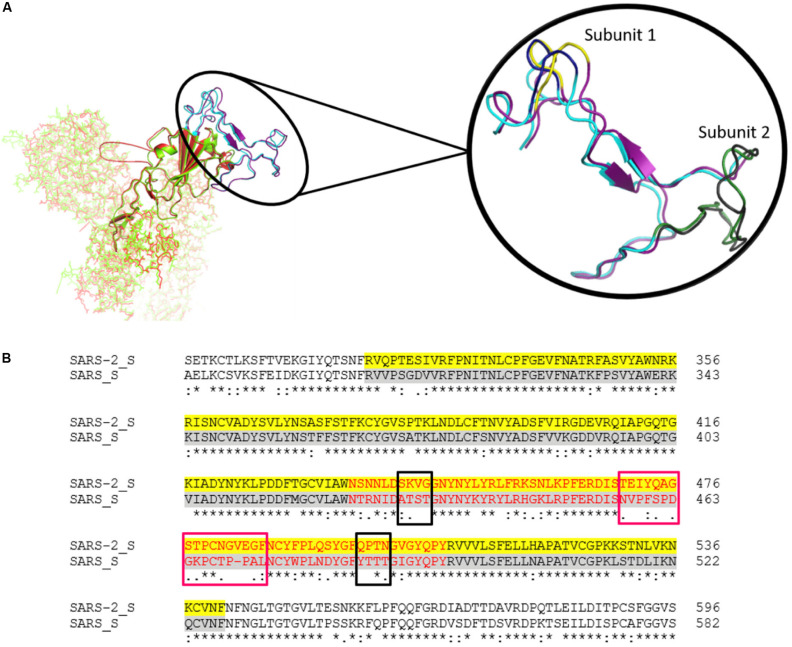
**(A)** The Spike protein structure of SARS-CoV-2 (green) compared to SARS-CoV (red). The RBD is represented by a cartoon, while the rest of the protein is represented by sticks. The highlighted part is the comparison of the RBM of SARS-CoV-2 (Purple) and SARS-CoV (Cyan). Magnified view of RBM shows both subunits. Subunit 1 of SARS-CoV-2 and SARS-CoV are highlighted in yellow and deep blue, respectively. Likewise, subunit 2 of SARS-CoV-2 and SARS-CoV are depicted in gray and forest green, respectively. **(B)** The sequence alignment of SARS-CoV-2 and SARS-CoV spike RBD. The yellow and gray shaded parts represent the RBD of both of the CoVs, and the RBM is represented in red. The conservancy and variation among subunit 1 (black boxes) and subunit 2 (pink boxes) are also shown in the alignment.

**FIGURE 5:**
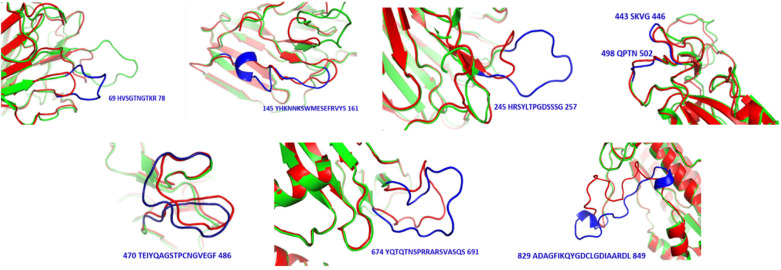
Structural differences between SARS-CoV (red) and SARS-CoV-2 (green). Sequences and positions of SARS-CoV-2 regions are highlighted in blue.

**FIGURE 6:**
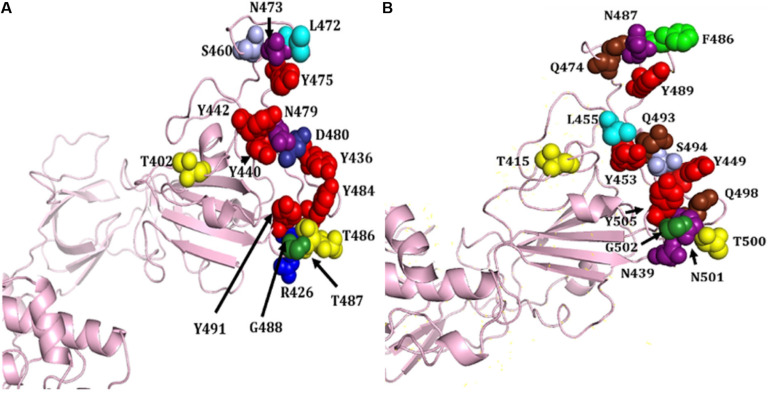
Structural representation of various attachment sites of S protein RBD to ACE2. **(A)** Known active sites of SARS-CoV. **(B)** Mapped putative active sites on SARS-CoV-2 S protein corresponding to SARS-CoV S protein. Different amino acids are shown in distinct colors, i.e., R, T, N, Y, L, S, Q, F, G, D, and N.

S proteins of SARS-CoV-2 and SARS-CoV share 75% sequence identity, and other strains of CoVs, MERS, HKU1, OC43, NL63, and 229E, share 35, 35, 37, 30, and 31% identity, respectively (Supplementary Figures S3, S9). Further, S protein mainly consists of receptor-binding domain (RBD) and receptor-binding motif (RBM), which are critically important for viral entry and attachment. The RBD of both SARS-CoV-2 and SARS-CoV shows high conservancy; however, it is important to notice that both of the subunits (S1 and S2) present in RBM show less conservancy, thus suggesting different modes and affinities to receptor binding and membrane fusion. The conservation and variation of RBD and RBM are shown in Figures 4A,B. The figure also depicts the receptor-binding S1 at amino-terminal and membrane fusion S2 subunits at carboxy-terminal along with RBD and RBM (Figures 4A,B). Further, some major structural differences between SARS-CoV and SARS-CoV-2 are depicted in Figure 5. Moreover, the active sites of S protein interacting with ACE2 are very critical for viral entry and transmission. We also analyzed and mapped the active sites, i.e., T402, R426, Y436, Y440, Y442, S460, L472, N473, Y475, N479, D480, Y484, T486, T487, G488, and Y491 of SARS-CoV S protein RBD on the SARS-CoV-2 S protein and marked the corresponding residues, which are structurally and sequentially conserved as putative active sites (Figure 6).

Based on the structural alignment, we found that amino acids at different positions, viz., T415, Y449, Y453, N487, Y489, T500, G502, and Y505, of SARS-CoV-2 S protein RBD remained the same, corresponding to the SARS-CoV S protein amino acids, i.e., T402, Y436, Y440, N473, Y475, T486, G488, and Y491, respectively (Figure 6). Furthermore, other amino acids, i.e., R and T at positions 426 and 487 of SARS-CoV was replaced by N at positions 439 and 501 of SARS-CoV-2, respectively. Likewise, L445 of SARS-CoV-2 replaced the aromatic amino acid Y442, Q at positions 474, 493, and 498 replaced S460, N479, and Y484 of SARS-CoV, respectively. The L472 and D480 of SARS-CoV were substituted by the aromatic amino acid F at positions 486 and S494 of SARS-CoV-2, respectively (Figures 4B, 6).

As S protein may be an ideal target for vaccine design and development and, to date, there is no licensed vaccine or drug available for the treatment of the infection (COVID-19), a peptide vaccine could be designed based on S protein subunit 1, relying on the fact that ACE2 is the SARS-CoV-2 receptor (Shang et al., 2020). We have also depicted the predicted potential B cell (linear and discontinuous) (Supplementary Figures S10, S11) and T cell (CD4^+^, CD8^+^, and CTL) vaccine candidates on S protein (Supplementary Figures S12, S13). The four predicted efficient linear B-cell epitopes present at different locations are as follows: 369-YNSASFSTFKCYGVSPTKLNDLCFT-393 (25 AA), 404-GDEVRQIAPGQTGKIAD YNYKLP-426 (23 AA), 206-KHTPINLVRDLPQGFS-221 (17 AA), and 656-NNSYECDIPI-666 (11 AA) (Supplementary Figure S10), and three discontinuous epitopes are shown on trimeric S proteins (Supplementary Figure S11).

Further, predicted CD4^+^ and CD8^+^ epitopes of SARS-CoV-2 are depicted on the S protein (Supplementary Figure S12). The predicted epitopes are present at the 231-IGIN ITRFQTLLAH-245 (14 AA) and 61-NVTWFHAIHV-70 (10 AA) positions, respectively. Likewise, predicted CTL epitopes, i.e., 746-STECSNLLL-754, 821-LLFNKVTLA-829, 1053-VV FLHVTYV-1061, 827-TLADAGFIK-835, 507-PYRVVVLSF-515, 712-IAIPTNFTI-720, 886-WTFGAGAAL-894, 327-VRFPN ITNL-335, 505-YQPYRVVVL-513, 1016-AEIRASANL-1024, and 898-FAMQMAYRF-906 of length 9-mer, are also represented on the SARS-CoV-2 S protein (Supplementary Figure S13).

We have focused on structure prediction and conservation analysis of distinct proteins of seven different CoVs, including SARS-CoV-2. Comparisons between different coronavirus proteins provided valuable information on protein evolution, conservation, and variations to strategically develop antiviral agents against different CoVs, specifically for SARS-CoV-2. We also provide mapping of putative binding sites of S protein and potential epitopes for the active development of anti-SARS-CoV-2 agents. Moreover, high conservation against different proteins of SARS-CoV and SARS-CoV-2 provides an opportunity for the repurposing of small molecules and inhibitors and the development of cross-protective vaccine and antiviral therapy.

## Conclusion

The ongoing infectious COVID-19 disease caused by SARS-CoV-2 has caused millions of deaths worldwide with no vaccine or therapeutic treatment to date to combat the deadly virus. To assist researchers in fighting SARS-CoV-2, we performed comprehensive meta-analyses and developed an integrative web-resource “CoronaVR.” Largely, we focus on and recommend potential anti-SARS-CoV-2 solutions, i.e., T-cell and B-cell epitopes for incorporation into vaccine formulations, siRNA-based therapeutic regimens, and diagnostic primers. These can be useful candidates for researchers working toward developing anti-SARS-CoV-2 solutions.

## Data Availability Statement

The datasets presented in this study can be found in online repositories. The names of the repository/repositories and accession number(s) can be found in the article/Supplementary Material.

## Author Contributions

MKu conceived, designed, and supervised this study. AG performed the data collection and curation and developed the web server. MKh, AR, VS, PP, KB, and AG performed the vaccine epitope analysis. Sh, CS, and Ba performed analysis of diagnostic primers, siRNAs, and glycosylation sites. AT performed miRNA analysis. AM performed sgRNA analysis. SC and AM performed phylogenetic analysis. Sa performed codon analysis. PK and MKa performed the protein structure prediction and analysis. AG, MKh, and MKu performed the data interpretation and wrote the manuscript. All authors contributed to the article and approved the submitted version.

## Conflict of Interest

The authors declare that the research was conducted in the absence of any commercial or financial relationships that could be construed as a potential conflict of interest.

## Footnotes

1 https://www.who.int/docs/default-source/coronaviruse/20200630-covid-19-sitrep-162.pdf?sfvrsn=e00a5466_2

2 http://tools.iedb.org/main/tcell/

3 http://www.cbs.dtu.dk/services/NetCTLpan/

4 http://tools.iedb.org/immunogenicity/

5 http://tools.iedb.org/CD4episcore/

6 http://crdd.osdd.net/raghava/ifnepitope/

7 http://tools.iedb.org/main/bcell/

8 http://tools.iedb.org/ellipro/

9 http://www.ddg-pharmfac.net/vaxijen/VaxiJen/VaxiJen.html

10 http://www.imtech.res.in/raghava/toxinpred/

11 http://www.ddg-pharmfac.net/AllerTOP/

12 http://tools.iedb.org/conservancy/

13 http://tools.iedb.org/population/

14 http://genomes.urv.es/CAIcal/

15 https://www.kazusa.or.jp/codon/

16 https://pymol.org/2/
