# Supplementary material for: CoronaVR: A Computational Resource and Analysis of Epitopes and Therapeutics for Severe Acute Respiratory Syndrome Coronavirus-2
Source: Front Microbiol. 2020 Jul 31;11:1858. doi: 10.3389/fmicb.2020.01858 (PMC7412965; doi:10.3389/fmicb.2020.01858)
Supplement: Supplementary file 1 [file Table_1.DOCX]

Additional file 1 (Supplementary Material)


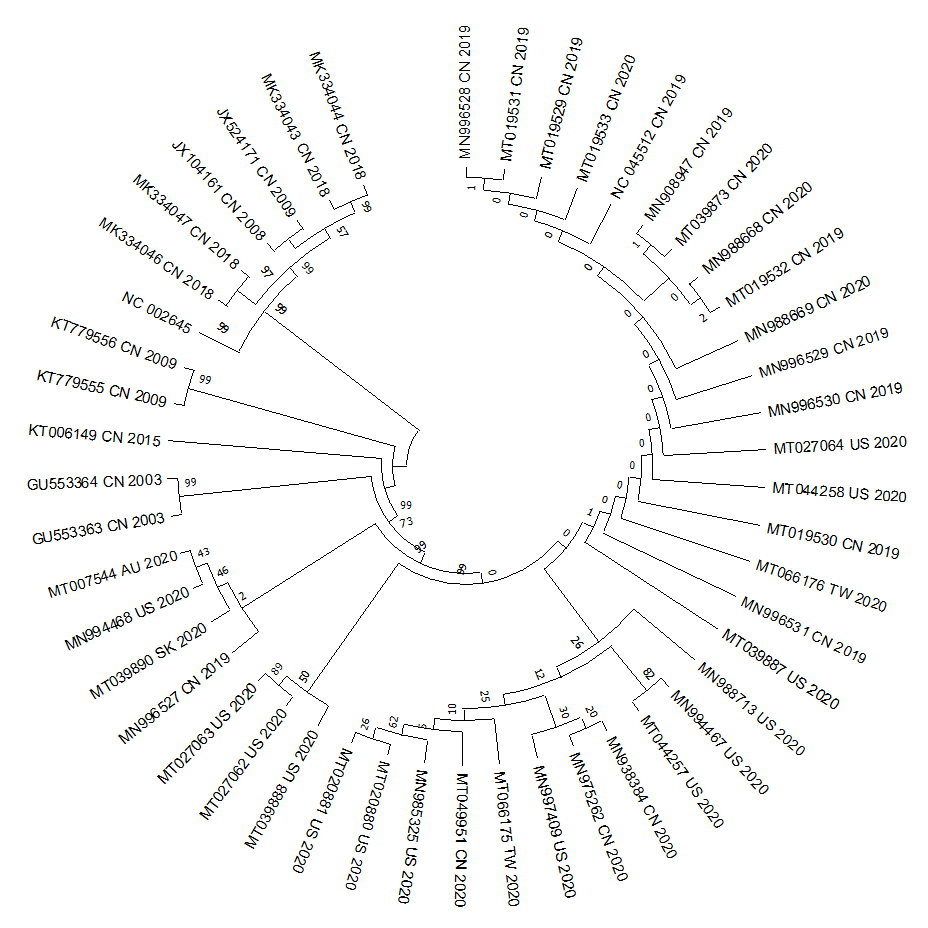


**Figure S1. Genome-based phylogeny.**

The evolutionary history was inferred by using the Maximum Likelihood method and General Time Reversible model. The tree with the highest log likelihood (-187941.24) is shown. The percentage of trees in which the associated taxa clustered together is shown next to the branches. Initial tree(s) for the heuristic search were obtained automatically by applying Neighbor-Join and BioNJ algorithms to a matrix of pairwise distances estimated using the Maximum Composite Likelihood (MCL) approach, and then selecting the topology with superior log likelihood value. A discrete Gamma distribution was used to model evolutionary rate differences among sites (5 categories (+G, parameter = 4.6816)). This analysis involved 48 nucleotide sequences. There was a total of 36494 positions in the final dataset. Evolutionary analyses were conducted in MEGA X.

**
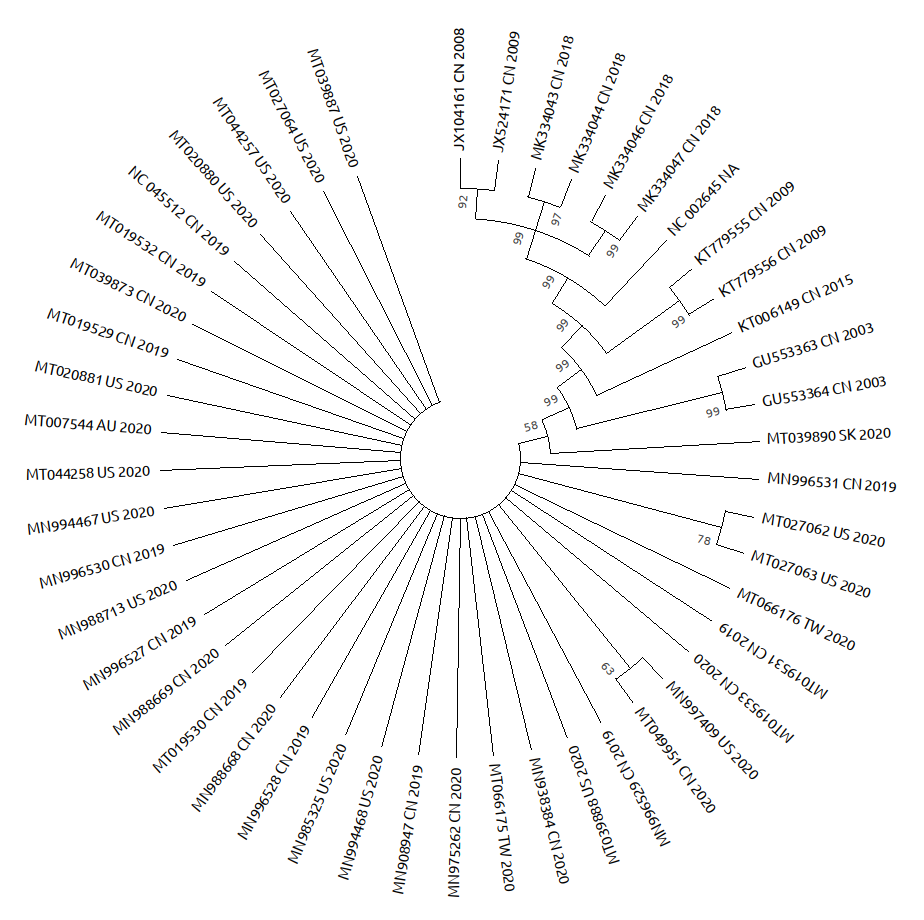
**

**Figure S2. Proteome-based phylogeny**.

Evolutionary analysis by Maximum Likelihood method. The evolutionary history was inferred by using the Maximum Likelihood method and Le_Gascuel_2008 model. The tree with the highest log likelihood (-65307.88) is shown. The percentage of trees in which the associated taxa clustered together is shown next to the branches. Initial tree(s) for the heuristic search were obtained automatically by applying Neighbor-Join and BioNJ algorithms to a matrix of pairwise distances estimated using a JTT model, and then selecting the topology with superior log likelihood value. A discrete Gamma distribution was used to model evolutionary rate differences among sites (5 categories (+G, parameter = 0.9414)). The tree is drawn to scale, with branch lengths measured in the number of substitutions per site. This analysis involved 48 amino acid sequences. All positions containing gaps and missing data were eliminated (complete deletion option). There was a total of 6075 positions in the final dataset. Evolutionary analyses were conducted in MEGA X.


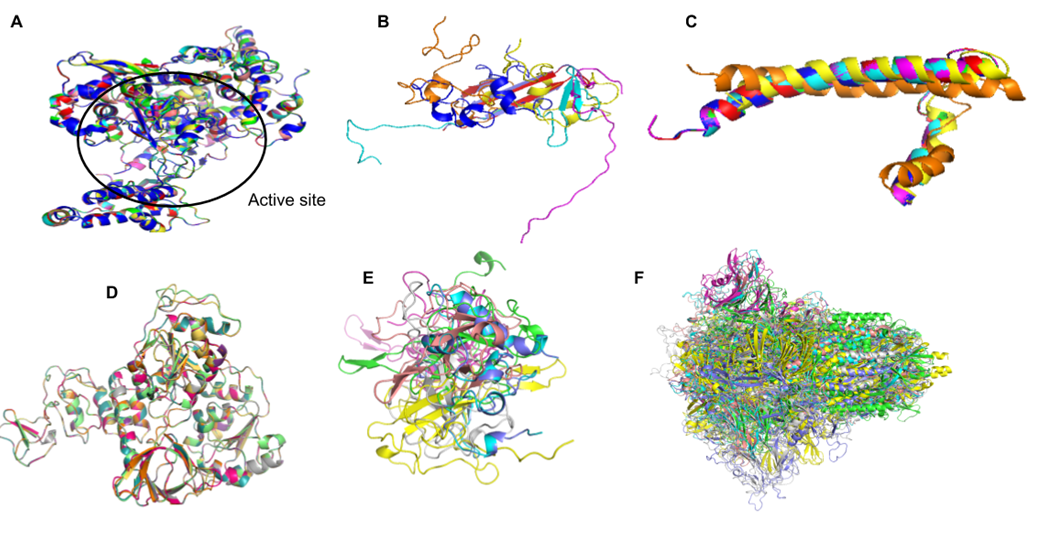


**Figure S3.** Three-dimensional structure comparison of different proteins of SARS-CoV-2 and other CoVs using PyMOL. (A) RdRp, (B) M, (C) E, (D) Helicase, (E) N, (F) S.


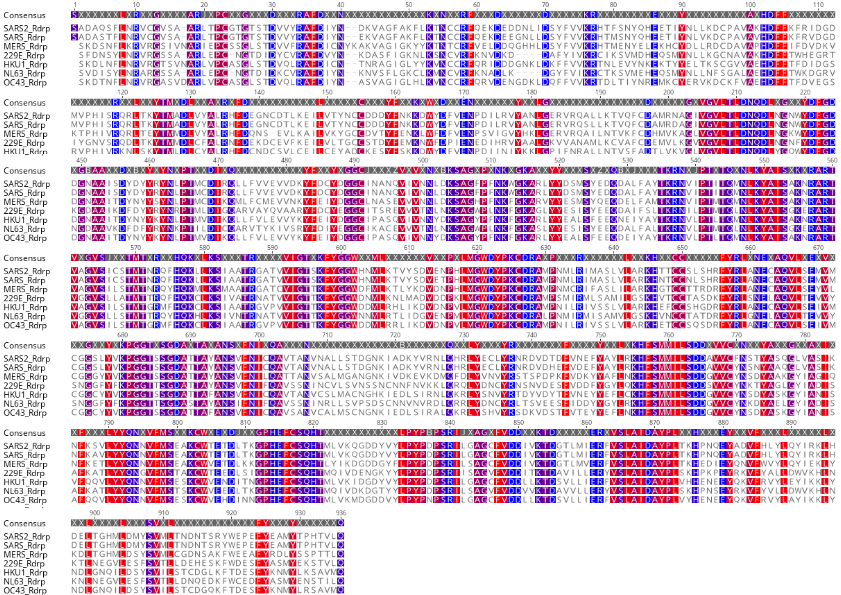
**Figure S4**. Sequence alignment of RNA-dependent RNA polymerase (RdRp) protein of SARS-CoV-2 with other coronaviruses


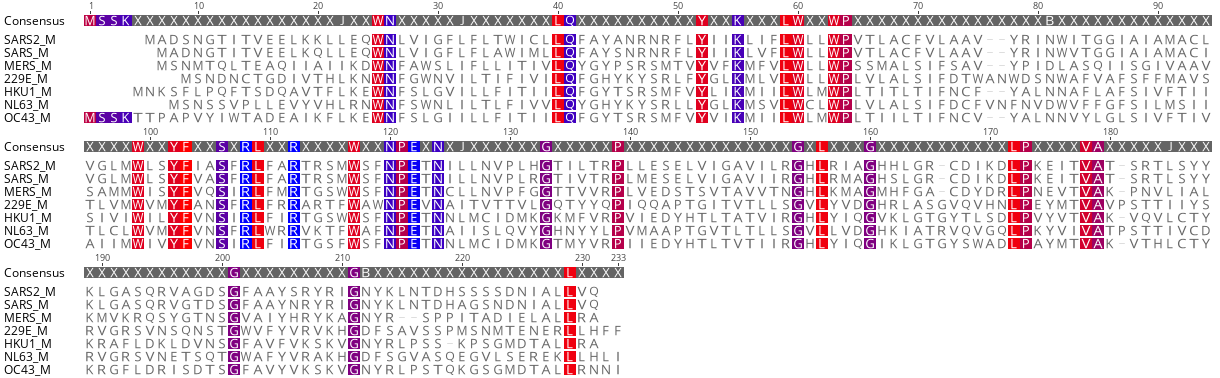


**Figure S5**. Sequence alignment of Membrane (M) protein of SARS-CoV-2 with other coronaviruses


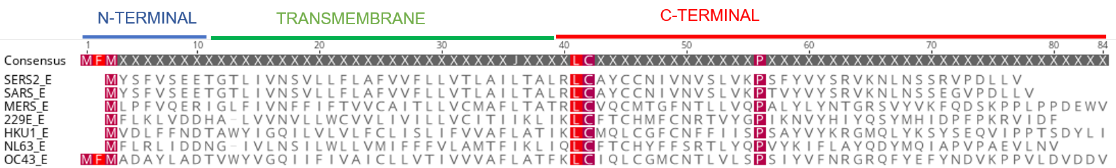


**Figure S6**. Sequence alignment of Envelope (E) protein of SARS-CoV-2 with other coronaviruses


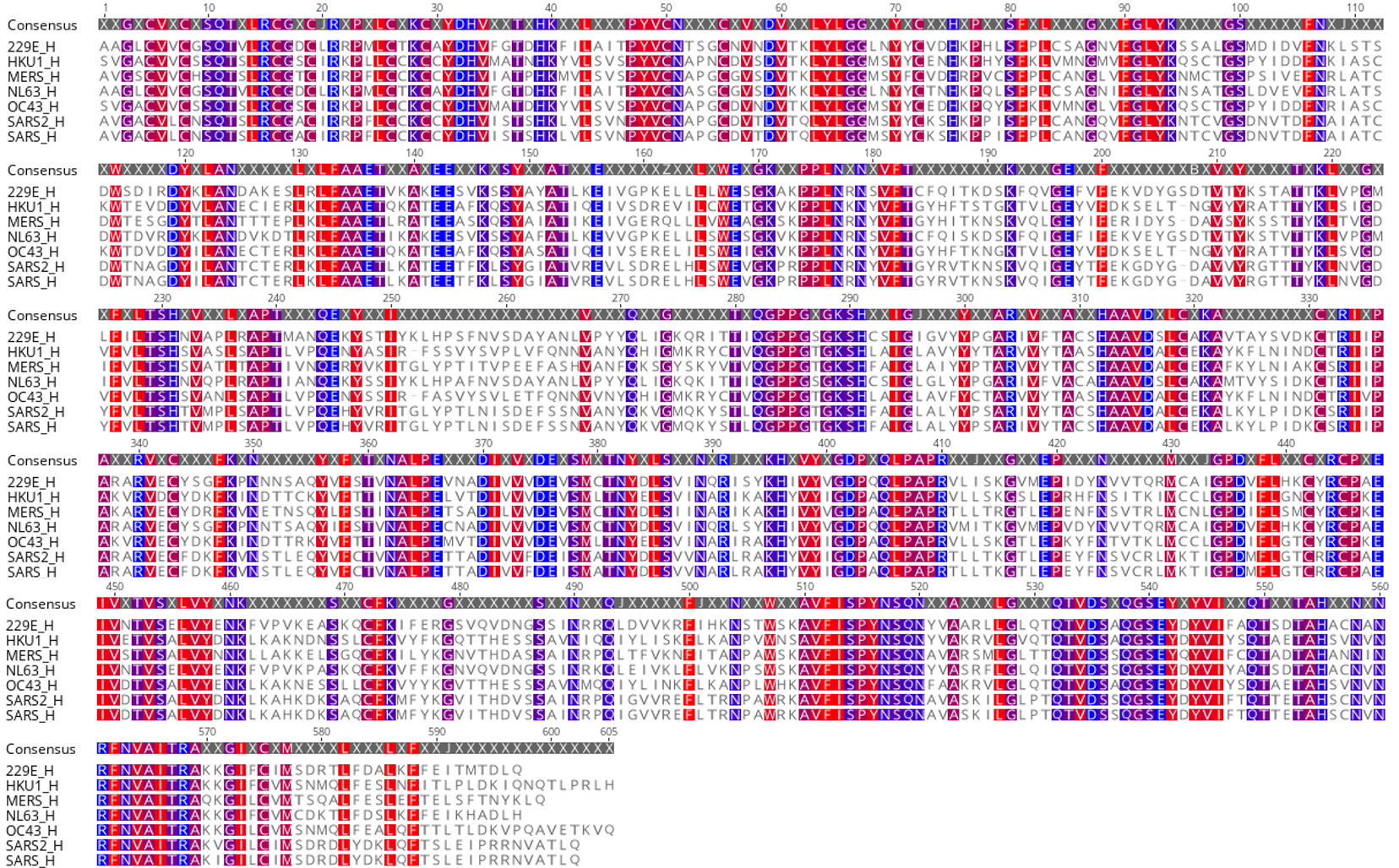


**Figure S7**. Sequence alignment of Helicase protein of SARS-CoV-2 with other coronaviruses


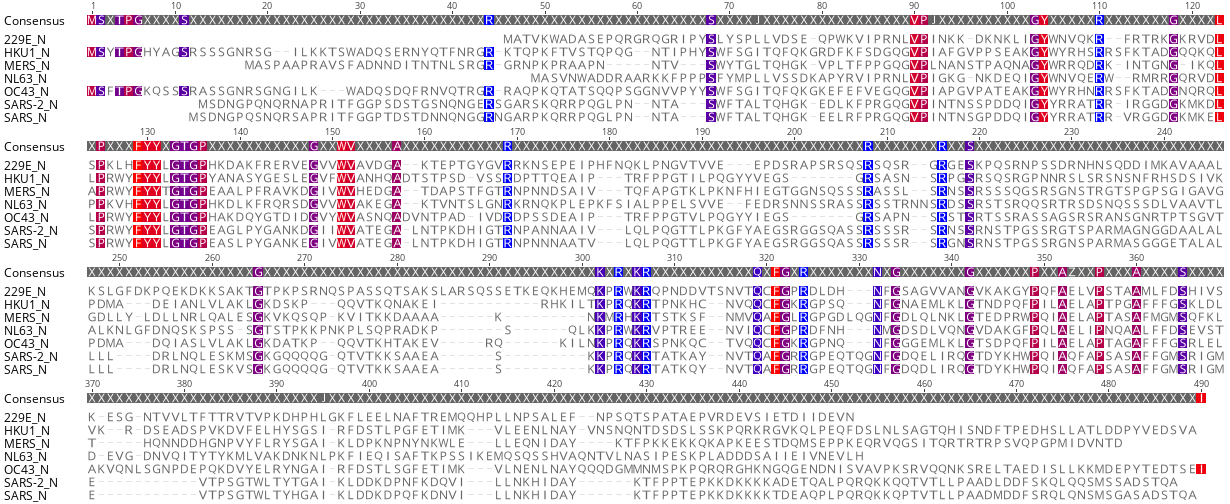


**Figure S8**. Sequence alignment of Nucleocapsid (N) protein of SARS-CoV-2 with other coronaviruses


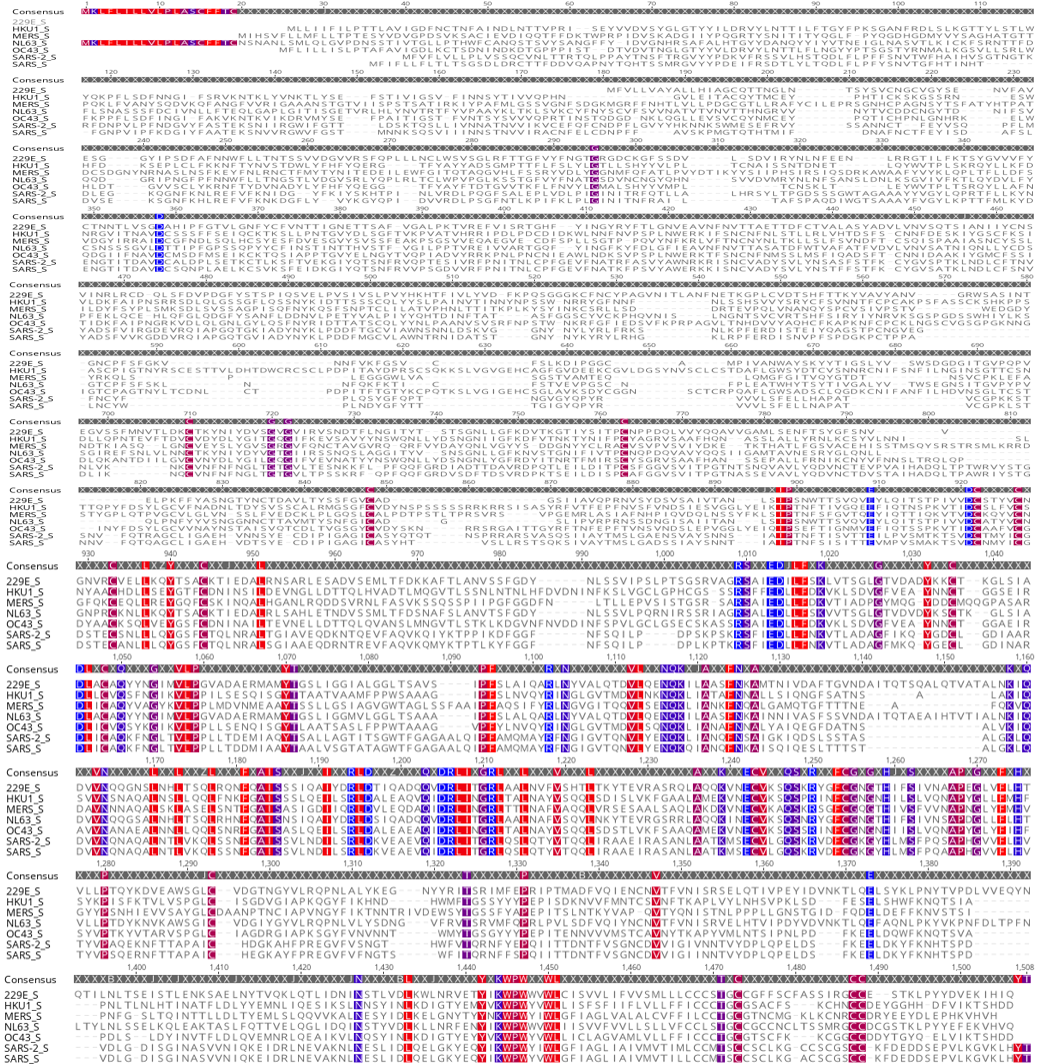


**Figure S9**. Sequence alignment of Spike glycoprotein (S) protein of SARS-CoV-2 with other coronaviruses


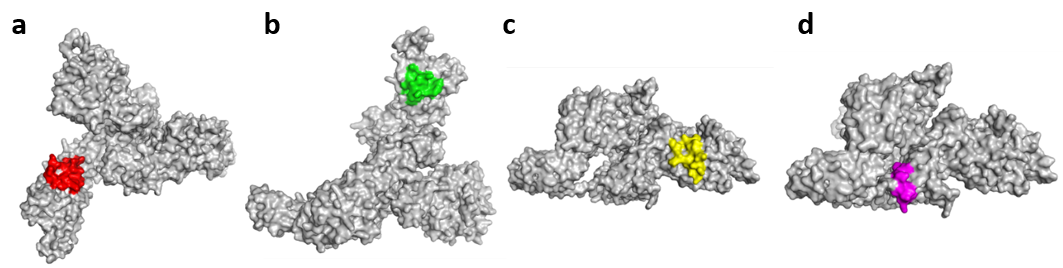


**Figure S10**. Representation of predicted efficient linear B-cell epitopes on the monomeric unit of spike protein of SARS-CoV-2. (a) 369-393, (b) 404-426, (c) 206-221, (d) 656-666.


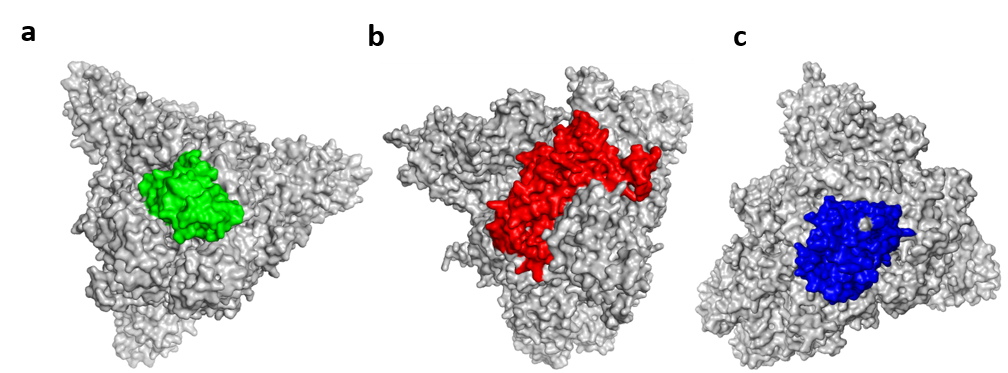


**Figure S11**. Representation of high scoring discontinuous B-cell epitopes. (a) from 703-1212 (156 residues, 0.801 score), (b) from 327-585 (224 residues, 0.752 score), (c) from 2-266 (183 residues, 0.738 score).


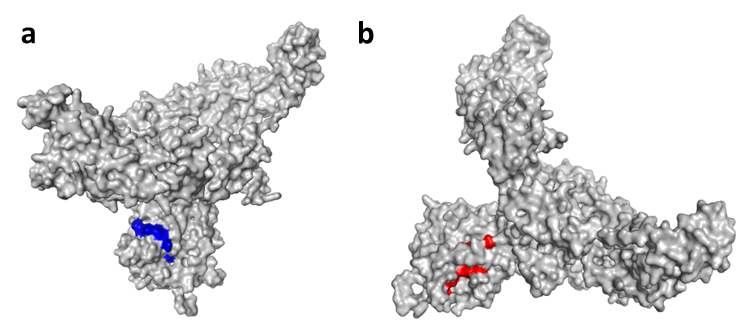


**Figure S12**. Representation of epitopes on the monomeric unit of SARS-CoV-2 spike protein. (**a**) CD4 at position 231-245 (**b**) CD8 at positions 61-70


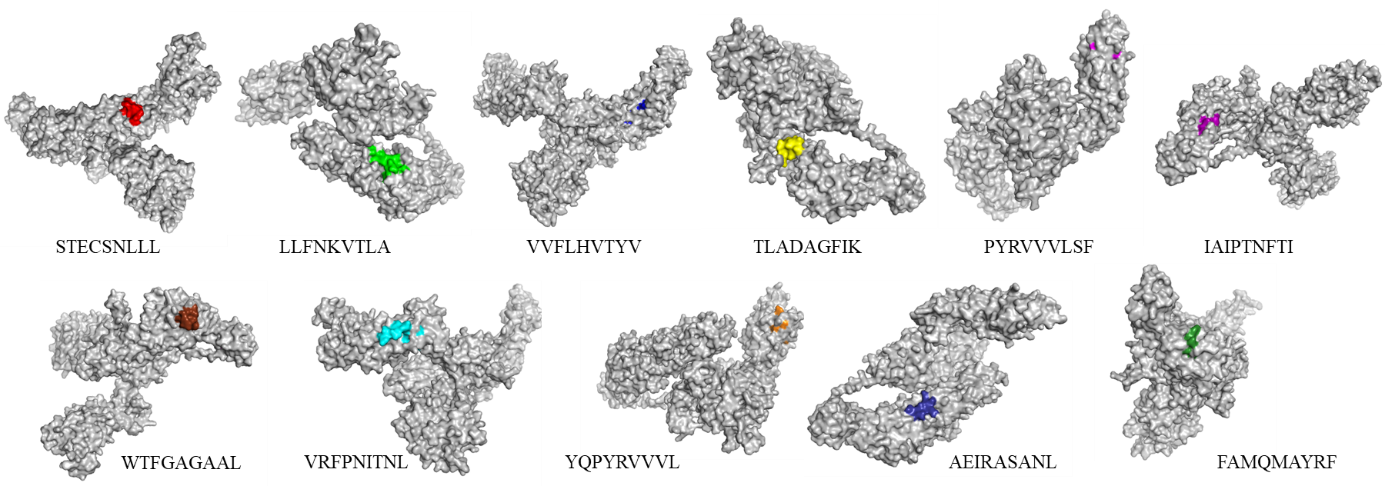


**Figure S13**. Representation of CTL epitopes on the monomeric unit of spike protein of SARS-CoV-2 with respective sequences
